# ChlamyNET: a *Chlamydomonas* gene co-expression network reveals global properties of the transcriptome and the early setup of key co-expression patterns in the green lineage

**DOI:** 10.1186/s12864-016-2564-y

**Published:** 2016-03-12

**Authors:** Francisco J. Romero-Campero, Ignacio Perez-Hurtado, Eva Lucas-Reina, Jose M. Romero, Federico Valverde

**Affiliations:** Departamento de Ciencias de la Computación e Inteligencia Artificial, Universidad de Sevilla, Reina Mercedes s/n, 41012 Sevilla, Spain; Instituto de Bioquímica Vegetal y Fotosíntesis, Universidad de Sevilla-CSIC, Americo Vespucio 49, 41092 Sevilla, Spain

**Keywords:** *Chlamydomonas reinhardtii*, green algae, gene co-expression networks, molecular systems biology, transcriptomics, RNA-seq, light-regulated transcription factors and transcriptional regulators

## Abstract

**Background:**

*Chlamydomonas reinhardtii* is the model organism that serves as a reference for studies in algal genomics and physiology. It is of special interest in the study of the evolution of regulatory pathways from algae to higher plants. Additionally, it has recently gained attention as a potential source for bio-fuel and bio-hydrogen production. The genome of *Chlamydomonas* is available, facilitating the analysis of its transcriptome by RNA-seq data. This has produced a massive amount of data that remains fragmented making necessary the application of integrative approaches based on molecular systems biology.

**Results:**

We constructed a gene co-expression network based on RNA-seq data and developed a web-based tool, ChlamyNET, for the exploration of the *Chlamydomonas* transcriptome. ChlamyNET exhibits a scale-free and small world topology. Applying clustering techniques, we identified nine gene clusters that capture the structure of the transcriptome under the analyzed conditions. One of the most central clusters was shown to be involved in carbon/nitrogen metabolism and signalling, whereas one of the most peripheral clusters was involved in DNA replication and cell cycle regulation. The transcription factors and regulators in the *Chlamydomonas* genome have been identified in ChlamyNET. The biological processes potentially regulated by them as well as their putative transcription factor binding sites were determined. The putative light regulated transcription factors and regulators in the *Chlamydomonas* genome were analyzed in order to provide a case study on the use of ChlamyNET. Finally, we used an independent data set to cross-validate the predictive power of ChlamyNET.

**Conclusions:**

The topological properties of ChlamyNET suggest that the *Chlamydomonas* transcriptome posseses important characteristics related to error tolerance, vulnerability and information propagation. The central part of ChlamyNET constitutes the core of the transcriptome where most authoritative hub genes are located interconnecting key biological processes such as light response with carbon and nitrogen metabolism. Our study reveals that key elements in the regulation of carbon and nitrogen metabolism, light response and cell cycle identified in higher plants were already established in *Chlamydomonas*. These conserved elements are not only limited to transcription factors, regulators and their targets, but also include the *cis*-regulatory elements recognized by them.

**Electronic supplementary material:**

The online version of this article (doi:10.1186/s12864-016-2564-y) contains supplementary material, which is available to authorized users.

## Background

The unicellular green alga *Chlamydomonas reinhardtii* (*Chlamydomonas*) is an important model organism for genomic and physiological studies in photosynthetic organisms. Due to its evolutionary position, it diverged from land-plants over a billion years ago, *Chlamydomonas* is considered a living representative of the photosynthetic organisms that gave rise to the *green lineage* [1]. Specifically, it has been used as a model organism to study the establishment, conservation and divergence of key biological processes in photosynthetic organisms such as the photoperiod response [2–4]. Recently, *Chlamydomonas* has attracted substantial interest for biotechnological applications in the context of bio-fuel and bio-hydrogen production [5–7]. The main advantage of using *Chlamydomonas* over higher plants is that it does not compete for agricultural land use. Additionally, *Chlamydomonas* posseses powerful genetic tools, metabolic versatility and a haploid genome. However, an important disadvantage is the lack of sufficient functional and regulatory characterization of the molecular mechanisms underpinning these processes with biotechnological interest [8].

In order to overcome this limitation its genome was sequenced and it is currently in an advanced curated state [1, 9]. The availability of its genome has facilitated the use of Next Generation Sequencing techniques, specially RNA-seq, in order to study its complete transcriptome. This has produced a massive amount of data from a variety of genotypes grown under relevant physiological conditions [10–16]. However, these studies remain fragmented without producing global insights into the organization and regulation of the *Chlamydomonas* transcriptome. The first steps towards the use of molecular systems biology methodologies to characterize the *Chlamydomonas* transcriptome has been taken [17–19]. Nevertheless, one of the most widely used tools for the integration and study of massive amounts of transcriptomic data, gene co-expression networks, have not yet been developed for *Chlamydomonas*, while gene co-expression networks have been used successfully in many other photosynthetic organisms [20–22].

Gene co-expression networks integrate fragmented transcriptomic data obtained in different studies in order to characterize patterns of coordinated gene expression at the whole transcriptome level. In gene co-expression networks nodes represent genes, being nodes connected by an edge if the corresponding genes are significantly co-expressed across appropriately chosen genotypes and physiological conditions [23]. Fundamental network concepts such as node degree, neighbourhood and clustering coefficient have important applications to unravel the organization and functioning of the represented transcriptome [24, 25]. The degree of a node, that is, the number of nodes connected to it, represents the number of genes co-expressed with the corresponding gene. Therefore, genes represented by nodes with high degrees are expected to be relevant in the transcriptome since their expression is coordinated with many others. The neighborhood of a node consists of genes co-expressed with the corresponding gene. This set of genes can be used as target genes candidates when the given gene is a transcription factor or potential regulator. The transcription factor binding sites that are responsible for the coordinated expression of genes can be identified by analyzing the significance of specific motifs in the promoters of co-expressed genes [26]. Additionally, Gene Ontology (GO) term enrichment over gene neighbourhoods can be applied to determine the potential biological processes that are carried out by the orchestrated expression of any given genes. In most gene co-expression networks the probability that a node is connected with *k* other nodes, *P* (*k*), follows a negative exponential distribution, *P* (*k*) ~ *k*^*-λ*^. This is the defining property of scale-free networks [27]. In scale-free networks most nodes are connected with few nodes, whereas there exists a small number of highly connected nodes called hubs that dominate the network dynamics [28]. Genes that correspond to hub nodes play a key role in the correct functioning of biological processes and, therefore, their mutation can lead to severely affected phenotypes and even lethality [29]. The clustering coefficient of a node meassures the tendency of nodes to group together around the given node, and when applied to gene co-expression networks, this concept indicates the tendency of genes to form highly co-expressed gene clusters. Scale-free networks with an average high clustering coefficient are called *small world networks* [28]. In this class of networks the existence of a clustering structure around hub nodes produces short paths that connect any pair of nodes. It has been often observed that biological co-expression networks are scale-free and small world networks [20, 25].

In this study we have developed ChlamyNET, a gene co-expression network and an associated web-based software tool that integrates the massive amount of RNA-seq data available for the *Chlamydomonas* transcriptome, see Additional file 1: Table S1. We have used this tool to study the organization and regulation of the algal transcriptome. ChlamyNET aims at becoming an enabling technology for researchers in the *Chlamydomonas* transcriptome, and in a wider perspective of alga transcriptomics, being the first tool of this kind existing at this date. Researchers can explore the neighbourhood of their genes of interest in ChlamyNET looking for potential targets or regulators. Additionally, our web tool can be used to determine GO terms related to biological processes, functions and components that are significantly present in the annotation of the neighbouring genes. Finally, we have used an independent experimental data set to cross-validate the predictive power of ChlamyNET.

## Results and discussion

### Network construction and topology

The high resolution provided by RNA-seq data and the diverse physiological conditions and genotypes analyzed allowed us to capture the co-expression relationships between genes in the *Chlamydomonas* transcriptome. In order to reduce the noise in our analysis, we only considered genes that showed significant changes in at least one comparison between a condition and its corresponding control. Data processing and selection of differentially expressed genes were performed as described in the methods section. Out of the 16624 predicted genes in the *Chlamydomonas* genome 13699 were differentially expressed in at least one of the conditions analyzed in this study. This represents 82*.*40 % of the algal genome, which shows that the analyzed conditions and phenotypes are diverse enough to capture the behaviour of most of the *Chlamydomonas* transcriptome.

**Fig. 1:**
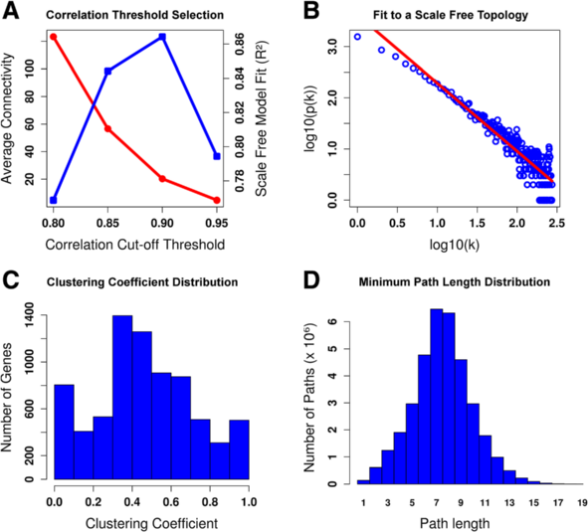
Network Construction and Topology Analysis. **a** Correlation Threshold Selection. The blue line shows that at the absolute value of correlation 0*.*90 the scale-free model fit exhibits a maximum. The red line shows that for increasing correlation thresholds the average connectivity of the network decreases, nonetheless for 0*.*90 it still presents a high value. According to this, the correlation threshold used to generate ChlamyNET was fixed to 0*.*90. **b** The degree distribution of a scale-free network follows an exponential negative distribution. The scale-free topology fit of ChlamyNET was computed using linear regression over the logarithmic transform of its degree distribution. **c** The clustering coefficient of a node or gene represents the degree of co-expression or correlation between its neighbours. Genes with a high clustering coefficient posses a high degree of co-expression or coordination among its co-expressed genes. ChlamyNET exhibits the high average clustering coefficient of 0*.*66. **d** ChlamyNET constitutes a *small world network*, that is a scale-free network with a high clustering coefficient. This is reflected in the fact that the average minimal path length between genes is 7*.*5

As described in the methods section, we used a range of absolute correlation thresholds to determine the co-expression level between the selected genes [30]. A compromise between the generation of a scale-free network and a high density network was established. We observed that for increasing correlation thresholds, the density of the network decreased, whereas the fit to the scale-free property increased until the cut-off value was too restrictive and the network started to deteriorate (Fig. 1a). Indeed, the scale-free model fit exhibits a maximum at a correlation value of 0*.*90 with an *R*^*2*^ equal to 0*.*86. According to this, we chose an absolute *Pearson correlation* threshold of 0*.*90 to consider that two genes are significantly co-expressed. The gene co-expression network generated for this threshold was called ChlamyNET.

ChlamyNET consists of 9171 genes or nodes exhibiting an overall of 139019 co-expression relationships or edges. ChlamyNET is composed of a major connected component consisting of 8443 genes (92*.*1 % of the entire network) and a multitude of small components consisting of a few genes. This global connectivity property of ChlamyNET is similar to previously constructed and analyzed networks from other organisms such as *Saccharomyces cerevisiae* [31] and *Arabidopsis thaliana* [32].

The scale-free property of ChlamyNET was corroborated by computing its degree distribution and checking that it follows a negative exponential distribution. Specifically, linear regression over the logarithmic transform of the degree distribution was used (Fig. 1b). Another topological property that we analyzed in ChlamyNET was the *clustering coefficient*, a measurement of the density of edges or co-expression relationships around genes. The distribution of the clustering coefficient in ChlamyNET was computed (Fig. 1c). The average clustering coefficient of ChlamyNET is 0*.*66 which is significantlly high when compared to random scale-free networks, see the methods section. This shows that ChlamyNET constitutes a non-random scale-free network with a high clustering coefficient. This type of networks are called *small-world networks* since the minimal path length between genes is short when compared to random scale-free networks [33]. These properties are common in gene co-expression networks [20, 32]. In the case of ChlamyNET the average minimal path length between genes or the *network diameter* is 7*.*5 (Fig. 1d). Therefore, on average any gene on ChlamyNET can be reached from another one through approximately seven gene co-expression relationships.

**Fig. 2:**
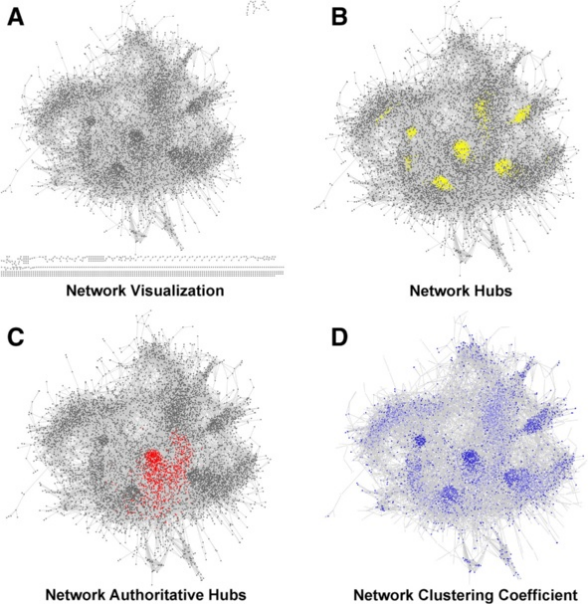
- Network Visualization, Hubs and Clustering Coefficient. **a** Graphical representation of ChalmyNET consisting of 9171 genes or nodes and 139019 co-expression relationships or edges. It is organized into a major connected component where most of the genes are located and a multitude of small components. **b** Network hubs. We have represented in yellow hub genes characterized by being co-expressed with a large number of other genes. Note that hub genes are located in specific regions of the network. **c** Authoritative hubs. Those hubs whose neighbours are highly connected, are mainly located at the core of the network. These authoritative hubs are represented in red. **d** The clustering coefficient of a gene meassures the degree of co-expression among its co-expressed genes. Genes with a high clustering coefficient are coloured in darker blue than those with a low clustering coefficient. Notice that regions of genes with a high clustering coefficient overlap with those where hubs are located

The topological properties of ChlamyNET (Fig. 2a), namely scale-free and small-world properties, imply that most genes in the *Chlamydomonas* transcriptome are co-expressed with only a few other genes. However, there exists a low number of genes that are co-expressed with a large number of other genes. These genes are called *hubs* and play a key role in the structure and functioning of gene co-expression networks [28]. We determined the first 1000 hubs in terms of their degree and highlighted them in ChlamyNET, observing that they are located in specific regions of the network (Fig. 2b). Nevertheless, the definition of hubs based solely on the number of genes it is co-expressed with has been found to be incomplete and the concept of *authoritative hub* has been introduced [34]. Following this line, in our context, an authoritative hub gene relevant to a biological process is not considered solely on the base that they have a large number of co-expressed genes. Additionally, since its co-expressed genes are involved in the same biological process they should in turn be co-expressed among themselves, establishing links in the network between them. These authoritative genes could then be responsible for bringing together genes potentially involved in a common biological process. We identified the first 1000 authoritative hubs using the *HITS algorithm* [34] and represented them in ChlamyNET (Fig. 2c). We observed that the most relevant authoritative hubs are located in the center of the network. Additionally, we observe that the location of regions with high clustering coefficient is not random. These regions substantially overlap with areas where hub genes are located (Fig. 2d). In order to determine whether or not these hub genes are involved in similar biological processes we performed a gene ontology (GO) term enrichment analysis (Table 1) based on orthology relationships with *Arabidopsis thaliana* and on the annotation of protein families and domains available in the Pfam database [35] as described in the methods section. This analysis revealed that the central part of ChlamyNET constitutes the core of the network where the most authoritative hub genes are located. These hubs interconnect key biological processes such as protein phosphorylation and response to light stimulus with carbon/nitrogen metabolism and transmembrane transport (Table 1). Protein kinases potentially involved in developmental processes such as *CrMEKK* (*g5375*), similar to the *Arabidopsis MEKK* gene *At5g57610*, and transcription factors possibly associated with circadian rythms and photoperiodic responses like *CrBbox1* (*Cre03.g182700*), similar to the *Arabidopsis COL1* gene *At5g15850*, are highly authoritative hub genes in the regulation of the *Chlamydomonas* transcriptome with more than 250 neighbours. Relevant enzymes in the carbon/nitrogen metabolism and transmembrane transport are also authoritative hub genes in the core of ChlamyNET indicating that their expression is highly regulated and coordinated with other biological processes. For example, the nitrate transporter *NRT2.3* (*Cre09.g396000*), nitrate reductase *NIT1* (*Cre09.g410950*) and starch phosphorylase *CrPHS1* (*Cre07.g336950*) are also co-expressed with more than 250 genes.

**Table 1:** Biological Process GO terms significally enriched in the 1000 most authoritative hub genes in ChlamyNET

GO term	Representative Genes	Potential Arabidopsis Ortholog	Number of neighbours
protein phosphorylation GO:0006468 (p-value 2.6 x 10^-11^)	Cre02.g108700 - Serine/Threonine Protein Kinase g2226 - VH1-Interacting Kinase Cre12.g537400 - ataurora Cre17.g742400 - Protein tyrosine kinase	At5g08160 At1g14000 At2g45490 At1g18160	245 253 266 93
transmembrane transport GO:0055085 (p-value 3 x 10^-7^)	Cre09.g396000 - Nitrate Transporter Cre10.g453400 - Mechanosensitive Channel of Small Conductance-like Cre01.g012700 - Gated Outwardly Rectifying K+ Channel	At1g12940 At5g12080 At5g37500	250 233 231
response to light stimulus GO:0009416 (p-value 2 x 10^-5^)	Cre03.g182700 - Bbox Protein Cre02.g118000 - Photolyase Cre12.g510200 - bZIP Protein g6302 - Constans-like Cre06.g295200 - Cryptochrome	At5g15850 At1g12370 At5g11260 At5g15840 At4g08920	259 182 133 58 57
carbohydrate metabolic process GO:0005975 (p-value 8 x 10^-5^)	Cre07.g336950 - Alpha-glucan phosphorylase Cre08.g362450 - Alpha Amylase g3160 - Isoamylase Cre04.g215150 - Soluble Starch Synthase	At3g46970 At1g69830 At2g39930 At5g24300	257 39 56 24
nitrogen compound metabolic process GO:0006807 (p-value 5.2 x 10^-5^)	Cre09.g410950 - Nitrate reductase Cre09.g410750 - Nitrite Reductase Cre03.g207250 - Glutamine synthetase	At1g37130 At2g15620 At5g35630	251 251 114

### Network clustering analysis and functional annotation

**Fig. 3:**
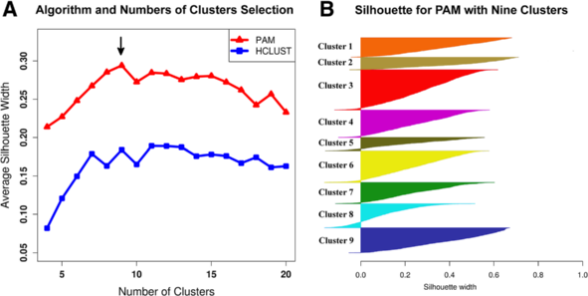
Selection of the Clustering Algorithm and Number of Clusters using as Criterion the Clustering Silhouette. **a** Algorithm and number of clusters selection. The absolute value of Pearson correlation coefficient between gene expression profiles was used as gene similarity measure to perform our clustering analysis. The performance of the clustering algorithms hierarchical clustering (HCLUST in red triangles) and partition around medoids (PAM in blue squares) were compared for different number of clusters ranging from 4 to 20 using the clustering silhouette. The highest silhouette value was reached for the PAM algorithm with nine cluster (marked with an arrow). **b** Silhouette for PAM with nine clusters. The silhouette of a clustering measures both the inter and intra cluster similarities. The best clustering silhouette obtained with the PAM algorithm for nine clusters is shown. Each horizontal line represents a gene in a given cluster. A high positive value indicates a gene with a high intra cluster similarity and a low inter cluster similarity. Whereas a negative value indicates a gene with a low intra cluster similarity and a high inter cluster similarity. Genes belonging to the same cluster are represented with the same colour. For each cluster from one to nine, the number of genes and its average silhouette are specified

The specific location of hub genes in regions exhibiting a high clustering coefficient may reflect an underlying structure in ChlamyNET relevant to physiological functions related to the *Chlamydomonas* transcriptome. In ChlamyNET we can observe distinct areas composed of genes with high degree and clustering coefficient that are in turn loosely connected through other genes with low degrees and clustering coefficients (compare Figs. 2b and d). This indicates the existence of relatively isolated groups of genes whose expression are highly coordinated and, hence, are potentially involved in the same biological processes. In order to corroborrate the existence of this underlying structure we applied clustering techniques over ChlamyNET using, as described in the methods section, the Pearson correlation coefficient between gene expression profiles as gene similarity measure. We compared the performance of the two most widely used clustering algorithms *hierarchical clustering (HCLUST)* and *partition around medoids (PAM)* for different number of clusters ranging from 4 to 20 clusters using the *silhouette*, a criterion that combines the minimization of inter-cluster similarity with the maximization of the intra-cluster similarity [36]. Our analysis concluded that the underlying structure of ChlamyNET is best described using nine clusters identified with the PAM algorithm (Fig. 3a) since this combination of clustering algorithm and number of clusters produced the highest silhouette value of 0*.*28 (Fig. 3b). This identified different clusters which we highlighted with different colour codes. Each gene cluster exhibits distinct expression profiles, see Additional file 2: Figure S1.

**Fig. 4:**
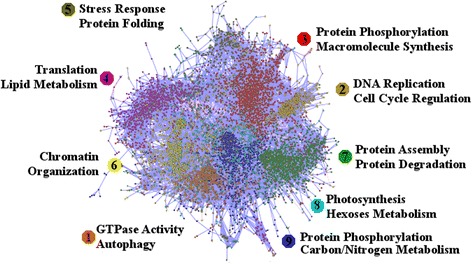
ChlamyNET Clustering and Cluster Functional Annotation. In ChlamyNET each node specifies a gene and an edge between genes represents that the corresponding gene expression profiles exhibit an absolute Pearson correlation coefficient value greater than 0*.*90. Therefore, edges represent co-expression relationships. Blue edges stand for positive correlation whereas pink edges stand for negative values. The nine different gene clusters are identified by numbers and different colours corresponding to the code in Fig. 3. Clusters are also annotated with the biological processes where the corresponding genes are potentially involved in

Since the genes in each cluster are co-expressed throughout the diverse physiological conditions integrated in this study they are likely involved in the same biological processes. In order to study the biological processes in which each gene cluster is involved we performed a Gene Ontology (GO) term enrichment over these clusters [37]. In order to overcome the limitations imposed by the sparse annotation of the *Chlamydomonas* transcriptome we combined GO terms obtained using orthology information with *Arabidopsis*, togheter with GO terms associated to protein families defined using conserved protein domains available from the Pfam database [35]. Since the used annotation is not based on experimental studies, the results obtained here should be taken as predictions that would need further experimental validation. This is precisely one of the main goals of GO term enrichment, namely the computational prediction of gene function to be subsequently corroborated using wet laboratory experimental work [38]. The results of our clustering and functional analysis are depicted in Fig. 4 and summarized in Table 2. The nine different gene clusters are identified with different colours and numbers following the code in Fig. 3b. In order to place our clustering analysis into a physiological, biochemical and metabolic context we used the tools and databases available from the *Plant Metabolic Network* [39]. Specifically, we used the extensive metabolic pathway information provided by *ChlamyCyc* [40] to identify the metabolic pathways contained in each cluster. In the following subsections, we present in detail four gene clusters and their functional annotation.

**Table 2:** Biological Process GO terms significally enriched in the clusters of the gene co-expression network ChlamyNET and the Metabolic and Signalling Pathways contained in each cluster

Cluster	Functional Annotation	Representative Genes	Metabolic/Signalling Pathways
Cluster 2 (Brown) 535 genes Silhoutte 0.44	DNA replication (GO:0006260)	Cre01.g015250 - POLD1 Cre16.g651000 - RFA1	Pyrimidine deoxyribonucleotides de novo biosynthesis pathway Cre16.g667850 - DUT Cre17.g715900 - THY Cre03.g190800 - TMPK
	Chromosome organization (GO:00051276)	Cre02.g086650 - SMC2 Cre12.g4 934 00 - SMC4	
	Regulation of Cell Cycle (GO:0010564)	Cre10.g466200 - CYCAB1 Cre03.g207900 - CYCA1	
Cluster 9 (Blue) 1058 genes Silhouette 0.40	protein phosphorylation (GO:0006468)	Cre17.g742400 - PTK17 Cre12.g537400 - CrAUR3	Starch Biosynthetic Pathway Cre04.g215150 - SSS Sucrose Biosynthetic Pathway Cre06.g283400 - SPP Nitrogen Assimilation Pathway Cre09.g410750 - NII1
	carbohydrate metabolic process (GO:0005975)	Cre08.g384750 - AMY Cre10.g444700 - SBE3	
	transmembrane transport (GO:0055085)	Cre09.g396000 - NRT2.3 Cre13.g564650 - MRS5	
Cluster 1 (Orange) 824 genes Silhouette 0.38	vesicle-mediated transport (GO:0016192)	Cre17.g728150 - Yky6 Cre16.g676650 - AP1G1	TAG Biosynthetic Pathway Cre02.g106400 - PDAT Phospholipid Biosynthetic Pathway Cre01.g035500 - PI3K Coenzyme A Biosynthetic Pathway Cre01.g048050 - COAB
	GTPase activity (GO:0043087)	Cre12.g532600 - CGL44 Cre07.g315350 - RABGAP	
	Autophagy (GO:0006914)	Cre09.g391500 - APG9	
Cluster 3 (Red) 1723 genes Silhouette 0.28	protein phosphorylation (GO:0006468)	Cre02.g145500 - PTK24 Cre12.g498650 - ALK3	TAG Biosynthetic Pathway g9572 - DGAT1 Hydrogen production Cre09.g396600 - HYDA2 MAP kinase cascade Cre10.g461150 - CrMAPKKK
	ribosome biogenesis (GO:0042254)	Cre12.g532550 - RPL13a Cre09.g400650 - RPS6	
	macromolecule biosynthesis (GO:0009059)	Cre03.g207250 - GLN4	
Cluster 4 (Purple) 1174 genes Silhouette 0.26	translation (GO:0006412)	Cre03.g199900 - EIF4E Cre02.g117900 - RH	tRNA Charging Pathway g2951 - TrpS Amino Acid Biosynthesis Cre03.g161400 - WSN2 Pentose Phosphate Non-oxydative Cre12.g511900 - RPE1 TAG Biosynthetic Pathway Cre03.g205050 - DGAT2
	RNA processing (GO:0006396)	Cre16.g653050 - SpoU Cre10.g421600 - ThrRS g4 679 - RNase P	
	lipid metabolism (GO:0006629)	Cre09.g397250 - FAD5 Cre06.g295250 - PAP	
Cluster 7 (Green) 909 genes Silhouette 0.25	protein complex assembly (GO:0006461)	g9912 - CSN5 Cre16.g663500 - CrRPN10	Aerobic Respiration Pathway Cre15.g638500 - CYC1 COP9 Signalling g11578 - CSN6
	response to misfolded protein (GO:0051788)	Cre06.g280850 - PSMB4 Cre12.g501200 - SKP1	
Cluster 6 (Yellow) 1351 genes Silhouette 0.24	chromatin organization (GO:0006325)	g11636 - HDA Cre13.g590750 - HTB37	Chromatin Remodelling Cre13.g591200 - HTB38 Cre13.g562400 - ABI3
	posttranscriptional regulation (GO:0010608)	g7250 - DCL	
Cluster 5 (Dark Green) 567 genes Silhouette 0.21	response to heat (GO:0009408)	Cre14.g617400 - HSP22F Cre08.g372100 - HSP70A	Stress Response Cre02.g098800 - ERP29 g9861 - TOR
	protein folding (GO:0006457)	g9881 - FKBP Cre01.g047700 - CYP40	
Cluster 8 (Turquoise) 1030 genes Silhouette 0.10	photosynthesis (GO:0015979)	Cre09.g412100 - PSAF Cre10.g44 04 50 - PSB28	Calvin Cycle Cre12.g554800 - PRK1 TCA Cycle Cre02.g143250 - IDH2
	hexose metabolic process (GO:0019318)	Cre17.g725550 - GLD1 Cre02.g093450 - FBA2	

#### Cluster 2, brown - DNA replication, chromosome organization and regulation of cell cycle

The most cohesive gene cluster is also the smallest one. The brown cluster is located in the periphery of ChlamyNET. It presents the highest silhouette value (0.44) in the network and contains 535 genes (Fig. 3b). Our GO term enrichment analysis reveals that this cluster is involved in cell cycle processes. Specifically, it is enriched in genes required for DNA replication (GO:0006260) such as DNA polymerase *POLD1* (*Cre01.g015250*), replication factor *RFA1* (*Cre16.g651000*) and origin recognition complex *ORC2* (*Cre03.g199400*); genes associated with chromosome organization (GO:0051276) like structural maintenance of chromosomes *SMC4* (*Cre12.g493400*) and *SMC2* (*Cre02.g086650*) and genes involved in the regulation of cell cycle process (GO:0010564) such as the cyclin A/B *CYCAB1* (*Cre10.g466200*) and the A-type cyclin *CYCA1* (*Cre03.g207900*).

The metabolic pathways located in this cluster produce DNA and RNA precursors such as the *pyrimidine deoxyribonucleotides de novo biosynthesis pathway*. For example, the formation of the DNA-specific end product dTTP starts with the hydrolyzation of dUTP to produce dUMP by the dUTP pyrophosphatase *DUT* (*Cre16.g667850*), followed by the reductive methylation of dUMP catalyzed by thymidylate synthase *THY* (*Cre17.g715900*) which yields dTMP. Finally, the thymidylate kinase *TMPK* (*Cre03.g190800*) catalyzes the first phosphorylation of dTMP leading to dTTP. These three enzymes are members of this cluster (Additional file 3: Figure S2 and Table 2).

#### Cluster 9, blue - protein phosphorylation, carbohydrate metabolic process and transmembrane transport

The blue cluster located in the center of ChlamyNET is enriched with hub genes according to a *p-value <* 2*.*2°10^−16^ obtained using Fisher's exact test. It is the second most cohesive cluster with a silhouette value of 0.40 and 1058 genes (Fig. 3b). The most significantly over-represented GO terms in this cluster are protein phosphorylation (GO:0006468) with genes such as the protein tyrosine kinases *PTK17* (*Cre17.g742400*) and ataurora *CrAUR3* (*Cre12.g537400*), carbohydrate metabolic process (GO:0005975) including genes like the alpha amylase *AMY* (*Cre08.g384750*), and transmembrane transport (GO:0055085) including genes coding for magnesium and cobalt transport protein *MRS5* (*Cre13.g564650*). An analysis of the metabolic context of this cluster reveals that core pathways in carbon and nitrogen metabolism are contained in it. Starch is the major reservoir of energy and carbon in photosynthetic organisms. The starch biosynthetic pathway constituted by the enzymes glucose-6-phosphate isomerase *PGI* (*Cre03.g175400*), phosphoglucomutase *PGM* (*g2899*), ADP glucose pyrophosphorylase *APL* (*Cre16.g683450*), starch synthase *SSS* (*Cre04.g215150*) and 1,4- -starch branching enzyme *SBE3* (*Cre10.g444700*) is entirely contained in this cluster. In *Chlamydomonas*, starch is degraded to hexoses during the dark period. The derived hexoses are then used in the sucrose synthesis pathway. Key enzymes in this pathway such as glyceraldehyde 3-phosphate dehydrogenase *GAP1* (*Cre12.g485150*) and sucrose phosphate phosphatase *SPP* (*Cre06.g283400*) are members of this cluster. The oxidative branch of the pentose phosphate pathway produces NADPH in the reactions catalized by glucose-6-phosphate dehydrogenase *GLD2* (*Cre08.g378150*) and 6-phosphogluconate dehydrogenase *GND1* (*Cre12.g526800*), enzymes coded by genes included in this cluster. NADPH is an important source of the reducing power required by many enzymes in central metabolic pathways. The anapleurotic pathway that fixes CO_2_ into oxaloacetate through the enzymes carbonic anhydrase *CAH8* (*Cre09.g405750*) and phosphoenolpyruvate carboxylases *PPC* (*g16646* and *g11831*) is also part of this cluster (Additional file 4: Figure S3). This pathway replenishes depleted Tricaboxylic Acid (TCA) cycle compounds that have been used for nitrogen assimilation or other tasks [41]. Inorganic and organic nitrogen assimilation pathways are included in this cluster (Additional file 4: Figure S3), including the nitrate transporter *NRT2.3* (*Cre09.g396000*), nitrite transporter *NAR1.4* (*Cre07.g335600*), nitrate reductase *NIT1* (*Cre09.g410950*) and nitrite reductase *NII1* (*Cre09.g410750*) yielding ammonia as a final product. In fact, these reductases need a molybdenum cofactor and the biosynthetic pathway for molybdenum cofactor constituted by the enzymes molybdopterin synthase adenylyltransferase *CNX* (*g10007*), cyclic pyranopterin monophosphate synthase *CNX2* (*Cre13.g602900*), molybdopterin synthase sulfurylase *MoaE* (*Cre07.g322250*) and molybdopterin molybdotransferase *MoeA* (*Cre10.g451400*) is entirely included in this cluster (Additional file 4: Figure S3). Therefore, not only the enzymes, but also the pathways leading to the synthesis of the cofactors needed for nitrate assimilation are tightly co-expressed in ChlamyNET.

#### Cluster 1, orange - intracellular transport, regulation of GTPase activity, autophagy and proteolysis

The orange cluster consists of 824 genes and is located in the periphery of ChlamyNET (Fig. 4). This cluster presents a high silhouette value of 0.38 (Fig. 3b). The GO term enrichment analysis indicates that genes within this cluster are significantly involved in processes related to intracellular transport to the endoplasmic reticulum and Golgi apparatus such as vesicle-mediated transport (GO:0016192). For instance, we can find genes coding for the endosomal R-SNARE protein *Yky6* (*Cre17.g728150*) and gamma1-Adaptin *AP1G1* (*Cre16.g676650*). Genes in this cluster are also significantly related to the regulation of GTPase activity (GO:0043087) such as those coding for the rab GTPase activator protein *CGL44* (*Cre12.g532600*) and *Rab/TBC* domain protein (*Cre07.g315350*). Autophagy (GO:0006914) and proteolyis (GO:0006508) are significant GO terms in this cluster with genes coding for the Autophagy related gene 9 *ATG9* (*Cre09.g391500*) and ubiquitin-conjugating enzyme E2 *UBC9* (*Cre16.g693700*). Therefore, the formation of this gene cluster suggests a connection between Rab GTPase activity and autophagy. Moreover, the positive regulation of Rab GTPase activity over autophagy has been shown in *Arabidopis* [42].

The metabolic analysis of this cluster suggests that it is involved in triacylglycerol (TAG) biosynthesis, the major lipid reserve in plants. Many unicellular microalgae accumulate large amounts of TAG under unfavorable conditions, such as the ones leading to autophagy [43]. TAG is produced from diacylglycerol (DAG) and different acyl donors. On the one hand, DAG can be synthesized from a 1,2-diacyl-sn-glycerol 3-phosphate and the enzyme phosphatidate phosphatase *PAH* (*Cre12.g506600*), a member of this cluster. On the other hand, phospholipids (major constituents of cellular membranes) are one of the possible donors for DAG to produce TAG. In this case, the enzyme phospholipid:DAG acyltransferase *PDAT* (*Cre02.g106400*) present in this cluster catalizes this reaction (Additional file 5: Figure S4). The 3-phosphoinositide biosynthesis pathway is also included in this cluster. Phosphoinositides are involved in phospholipid biosyntehsis as well as membrane trafficking, biological processes over-represented in this cluster. The key enzymes in this pathway are phosphatidylinositol-3-kinase *PI3K* (*Cre01.g035500*), phosphatidylinositol 4-kinase *PIK1* (*Cre05.g245550*), phosphatidylinositol-4-phosphate 5-kinase *PIP5K3* (*g9964*) and inositol 5-phosphatase *SAC1* (*Cre12.g537500*) which are also located in this cluster (Additional file 5: Figure S4). Other important lipid metabolic reactions are the activation and deactivation of lipids achieved by the ligation or removal of acyl-CoA. These reactions are catalized by the enzymes long-chain-fatty-acid-CoA ligase *LACS* (*Cre03.g182050*) and acyl-CoA thioesterase *ACOT* (*Cre01.g037350*) respectively, both members of this cluster. In these reactions the common acyl carrier Coenzyme A is required, and so, key enzymes in its biosynthesis such as ketopantoate hydroxymethyltransferase *PAN2* (*Cre12.g508550*), phosphopantothenate-cysteine ligase *COAB* (*Cre01.g048050*) and phosphopantothenoylcysteine decarboxylase *COAC* (*Cre10.g423450*) are also co-expressed in this cluster (Additional file 5: Figure S4).

#### Cluster 3, red - protein phosphorylation, translation, ribosome biogenesis and macromolecule biosynthetic process

The red cluster expands from the periphery of ChlamyNET to its core (Fig. 4). Somehow this cluster serves as an interface between the blue cluster (hub genes involved in protein phosphorylation, carbohydrate metabolic process and transmembrane transport) and the brown cluster (cell cycle processes). This cluster is the largest one including 1723 genes and presenting a moderate silhouette value of 0.28 (Fig. 3b). According to the GO term enrichment analysis, genes in this cluster are significantly involved in diverse biological processes. The three most significant processes are protein phosphorylation (GO:0006468) including genes such as the mitogen activated protein kinase *PTK24* (*Cre02.g145500*) and aurora-like kinase *ALK3* (*Cre12.g498650*); translation (GO:0006412) and ribosome biogenesis (GO:0042254) with genes coding for ribosomal proteins L13 *RPL13a* (*Cre12.g532550*) and S6e *RPS6* (*Cre09.g400650*). The next significant biological process is macromolecule biosynthetic process (GO:0009059) with genes such as the glutamine synthetase *GLN4* (*Cre03.g207250*).

The analysis of the metabolic pathways included in this cluster identified the synthesis of triacylglycerol using exclusively as acyl donors galactolipids produced by glycolipid desaturation. The diacylglycerol O-acyltransferase *DGAT1* (*g9572*) and monogalactosyldiacylglycerol synthase *FAD6* (*Cre13.g590500*) are thus included in this cluster (Additional file 3: Figure S2). Although no other metabolic pathway is fully represented in cluster 3, isolated key enzymes for carbon xation, hydrogen production and oxidation such as rubisco *RBCS2* (*Cre02.g120150*) and iron hydrogenase *HYDA2* (*Cre09.g396600*) are co-expressed within this cluster. In fact, our study suggests that this cluster is involved in signalling and transcription control rather than in metabolism. Several serine/threonine protein kinases are included in this cluster. The genes *CrAUR1* (*Cre16.g669800*) and *ALK3* (*Cre12.g498650*) exhibit a high sequence similarity with the and Aurora kinases in *Arabidopsis AUR1* (*At4g32830*) and *AUR3* (*At2G45490*) respectively. It has been described that the diversification of plants and aurora kinases predates the origin of land plants [44]. Here we show that this diversification may be already present in *Chlamydomonas*. These kinases have been shown to play a key role in cell cycle related signal transduction pathways in *Arabidopsis*. Several other genes similar to cyclin-dependent protein kinases are located in this cluster such as *CDKI1* (*Cre12.g494500*) and *CrMAPKKK* (*Cre10.g461150*). Cyclin-dependent protein kinases play crucial roles in the progression of the cell cycle in eukaryotes. *CDKI1* (*Cre12.g494500*) exhibits a high sequence similarity with the *Arabidopsis* gene *CAK4* (*At1g66750*), which is known to be involved in the activation of cell proliferation [45]. While *CrMAPKKK* (*Cre10.g461150*) is highly similar to the *Arabidopis* gene *MEKK1* (*At4g08500*). Additionally, other genes in this cluster such as *g16721*, present a high similarity with the *Arabidopsis* Mitogen Activated Protein (MAP) kinase *MAPKKK6* (*At3g07980*). The co-expression of these genes suggests that MAP kinase cascades are regulated not only at the posttranslational level but also at the transcriptional level in *Chlamydomonas*.

As it will be described in detail in the next section, this cluster is also significantly enriched in transcription factors. Several GATA transcription factors such as *g7394*, *Cre05.g242600* and *Cre08.g378800*; bZIP transcriptions factors like *Cre10.g438850* and *Cre12.g489000* and the single DOF and CO-like transcription factors in *Chlamydomonas CrDOF* (*Cre12.g521150*) [46] and *CrCO* (*g6302*) [2] are members of this cluster.

A detailed description of the rest of clusters and their functional annotation is available for further exploration at the web page http://viridiplantae.ibvf.csic.es/ChlamyNet/. These results aim at providing researchers in the functional annotation of the *Chlamydomonas* transcriptome with a solid ground to design specific and targeted experimental studies to validate or refute the predictions produced in this clustering analysis.

### Transcription factors and transcriptional regulators in ChlamyNET

In the previous section we performed a functional annotation of the different gene clusters identified according to GO term enrichment and metabolic pathways analysis. In this section, we further investigate the regulatory aspects of the *Chlamydomonas* transcriptome using ChlamyNET.

**Fig. 5:**
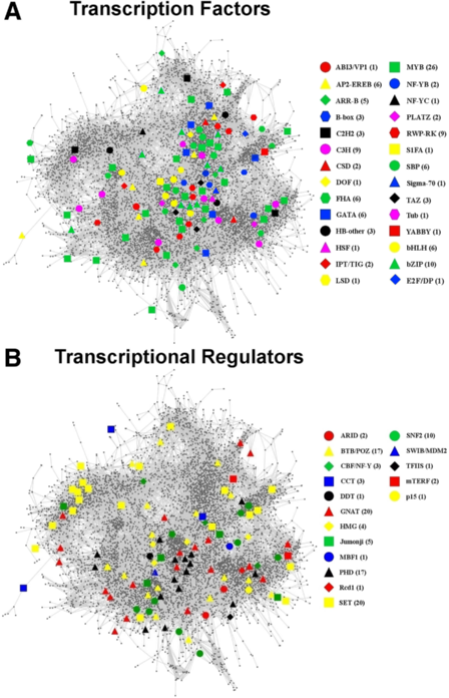
Location of Transcription Factors and Transcriptional Regulators in ChlamyNET. **a** Transcription Factors in ChlamyNET. We identified 118 different TFs classified into 28 different families represented using symbols with different colours and shapes. The distribution of the TFs over the clusters of ChlamyNET is not uniform. Clusters 9 (blue) and 3 (red) are enriched in TFs according to p-values of 2*.*62°10 ^−3^ and 2*.*37°10 ^−3^ obtained using Fisher's exact test. **b** Transcription Regulators in ChlamyNET. We identified 109 different TRs classified into 17 different families represented using symbols with different colours and shapes. The distribution of the TRs over ChlamyNET is uniform. Our analysis based on the Fisher's exact test did not identify any cluster significantly enriched in Trs

One of the most important processes involved in cellular response to internal and external stimuli is transcription or gene expression. This is a highly regulated process carried out by transcription factors and transcriptional regulators. Transcription factors (TFs) bind to specific *cis*-elements in the promoters of genes to activate or repress their transcription directly. On the other hand, transcriptional regulators (TRs) are involved in gene expression control but do not bind directly to gene promoters. Transcriptional regulators modulate gene expression by interacting with transcription factors, remodeling chromatin or other indirect mechanisms [47]. A genome-wide identification and classification of transcription factors and transcriptional regulators in *Chlamydomonas* has been previously performed [48]. This classification is available on the web portals and databases PlantTFDB (http://planttfdb.cbi.pku.edu.cn/index.php?sp=Cre) [49] and PlnTFDB (http://plntfdb.bio.uni-potsdam.de/v3.0/index.php?sp id = CRE4) [50]. Using this classification, we identified 118 TFs and 109 TRs in ChlamyNET, which constitute 2*.*48 % of the total number of genes in the network (Fig. 5). These TFs and TRs are classified, according to their protein domains, into 28 and 17 gene families respectively. The TFs seem not to be randomly distributed over the clusters of ChlamyNET (Fig. 5a), whereas the TRs distribution seems to be more uniformly distributed over the net (Fig. 5b). In order to asses the statistical significance of the distribution of TFs and TRs over the clusters in ChlamyNET, we performed an enrichment analysis based on Fisher's exact test. Indeed, no cluster was significantly enriched in TRs whereas the blue and red clusters were significantly enriched in TFs with p-values of 2*.*62°10^−3^ and 2*.*37°10^−3^ respectively. This complements the evidence produced by our analysis of the location of authoritative hub genes in ChlamyNET and about the key role played by the blue and red clusters in the regulation of the *Chlamydomonas* transcriptome under the conditions analyzed in this study.

**Fig. 6:**
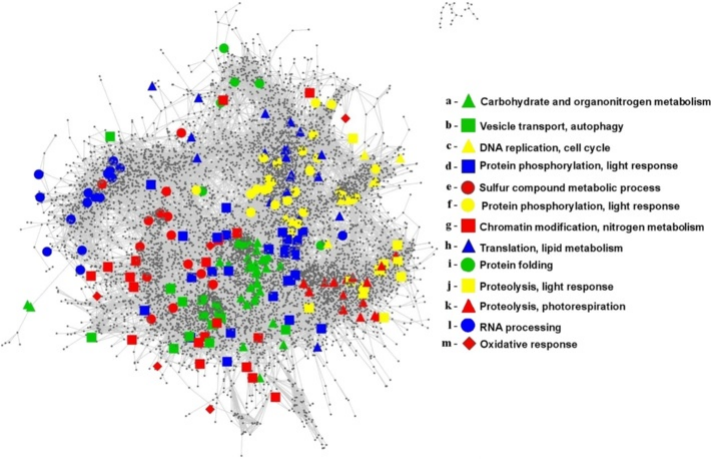
Transcription Factors and Transcriptional Regulators Clustering and Functional Annotation. According to the similarity between their gene expression profiles the TFs and TRs in ChlamyNET can be classified into 13 different groups identified by different symbols, colours and letters. The analysis of the GO terms overrepresented in the neighbourhood of each group suggest the biological processes that they might be regulating

In our analysis, instead of classifying genes according to their sequence as previously described [48], we have studied their co-expression patterns in order to determine groups of TFs and TRs that could exert their function over target genes in a coordinated way. Similar to the previous section we applied the two most widely used clustering algorithms, hierachical clustering and partition around medoids, taking as distance among genes the correlation between their expression profiles. According to the silhouette criterion, the best description of the co-expression patterns among TFs and TRs is obtained with thirteen different groups identified with the partition around medoids algorithm (Fig. 6).

In order to determine the biological processes regulated by each group we applied GO term enrichment analysis over the genes directly linked to the corresponding TFs and TRs. Additionally, we performed a transcriptional factor binding site (TFBS) enrichment analysis over the promoters of these genes as described in the Methods section. In Tables 3 and 4 we show a summary of the results about the GO terms and TFBS significantly enriched in the genes directly linked to the TFs and TRs in each group.

**Table 3:**
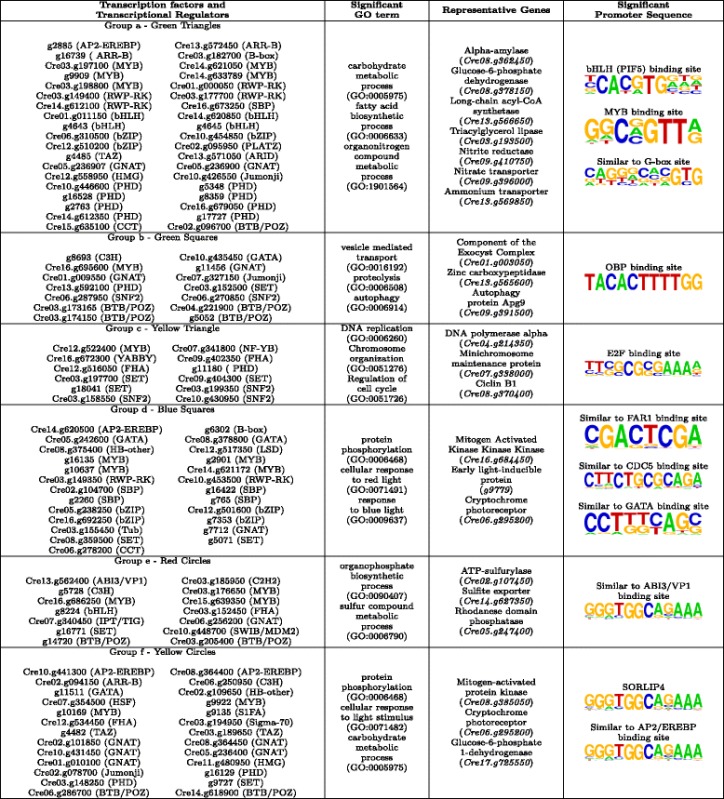
Biological processes and transcription binding sites significantly over-represented in the neighbourhood of the TFs and TRs groups in ChlamyNET

**Table 4:**
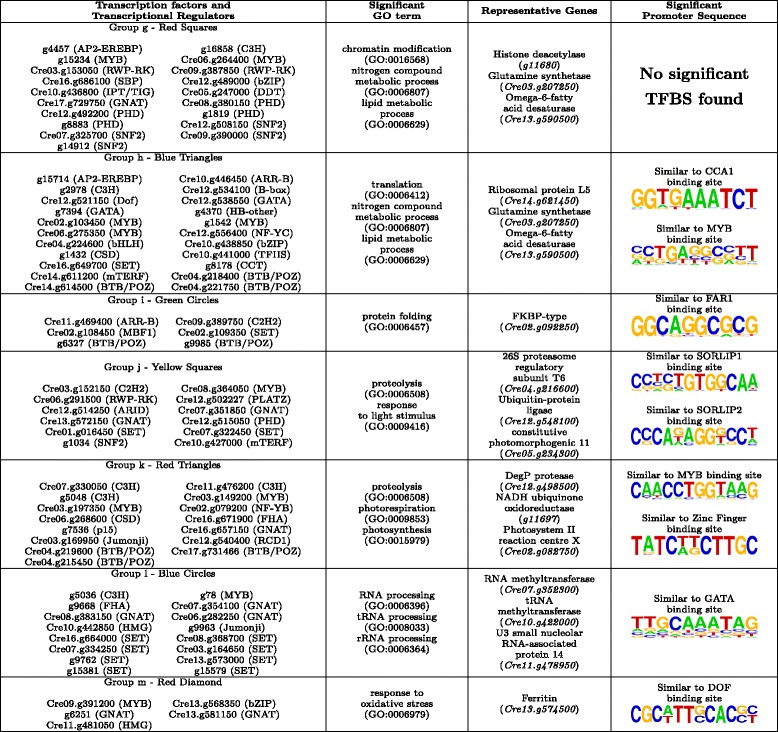
Biological processes and transcription binding sites signifcantly over-represented in the neighbourhood of the TFs and TRs groups in ChlamyNET

Herein we present in detail the results of our analysis over three groups of TFs and TRs of special interest. We discuss the conservation of their function and binding sites when compared to their putative orthologs in higher plants. The results for the remaining groups of TFs and TRs identified in our analysis are available at the web page http://viridiplantae.ibvf.csic.es/ChlamyNet/.

#### Core metabolic regulation, group a

This constitutes a numerous group of TFs and TRs including 38 members. They are identified in Fig. 6 using green triangles. The TFs and TRs in this group are included in the cluster 9 (blue) at the center of the network where most authoritative hub genes and carbon/nitrogen core metabolic pathways are located. These TFs and TRs seem to be of key importance in the regulation of the *Chlamydomonas* transcriptome under the conditions of our study since they are co-expressed on average with 87*.*97 other genes. Some highly authoritative hub genes in ChlamyNET are members of this group such as the B-box TF *CrBbox1* (*Cre03.g182700*), the bHLH TFs *g4643* and *Cre01.g011150*, the SBP TF *Cre16.g673250*, the RWP-RK TF *NIT2* (*Cre03.g177700*) and the MYB TF *Cre03.g197100*. These TFs present a normalized authoratitave hub score higher than 0*.*8. GO term enrichment analysis over the genes directly linked to the TFs and TRs in this group suggests that they are mainly involved in core metabolism regulation and light response. Several GO terms related with metabolic processes are significantly enriched such as carbohydrate metabolic process, fatty acid biosynthetic process and nitrogen compound metabolic process. Representative genes in this group are the alpha-amylase *AMA2* (*Cre08.g362450*), the long-chain acyl-CoA synthetase *LACS2* (*Cre13.g566650*) and the nitrite reductase *NII1* (*Cre09.g410750*), respectively.

Four bHLH transcription factors, *Cre01.g011150*, *Cre14.g620850*, *g4643* and *g4645*, out of the 12 recognized members of this family in *Chlamydomonas*, are members of this group. Only bHLH *Cre14.g620850* has similarity with genes present in higher plants. Specifically, its putative *Arabidopsis* ortholog is *PAR1* (*At1g69010*) that has been shown to be involved in light response [51]. The rest show similarity with other bHLH genes present only in chlorophyceae. A bHLH binding site was found to be significantly present over the genes co-expressed with the TFs and TRs of this group (Table 3). This suggests that the binding site of bHLH TFs is conserved accross the green lineage. Several genes involved in carbohydrate and nitrogen metabolism contain this binding site in their promoters, for instance the glucose-6-phosphate dehydrogenase *GLD2* (*Cre08.g378150*) and the ammonium transporter *AMT4* (*Cre13.g569850*).

Three bZIP TFs out of the 19 identified in the *Chlamydomonas* genome, *Cre10.g454850*, *Cre12.g510200* and *Cre06.g310500*, are members of this group. Genes *CrHY5* (*Cre12.g510200*) and *CrHYH* (*Cre06.g310500*) present a high similarity with the *Arabidopsis* genes *HY5* (*At5g11260*) and *HYH* (*At3g17609*) respectively. These TFs are known to bind to G-box sequences to regulate light response and metabolism in *Arabidopsis* [52, 53]. GO term and TFBS enrichment analysis suggest that this mechanism is already present in *Chlamydomonas*, since a sequence highly similar to the G-box has been found to be significantly present in the genes co-expressed with these two *Chlamydomonas* genes (Table 3).

*Cre13.g572450* and *g16739* that code for two ARR-B TFs and *CrBbox1* (*Cre03.g182700*), that codes for a B-box TF, are present in this group. These genes exhibit high similarities with the *Arabidopsis* genes *RR14* (*At2g01760*), *TOC1* (*At5g61380*) and *COL1* (*At5g15850*), respectively. They have in common a CCT domain at the carboxyl end that directly binds to DNA [54] that was found to be present in the *CrCO* (*g6302*) gene [2]. These genes are known to be involved in light response and circadian rythms in *Arabidopsis* [55, 56]. These functions seem to have been established already in *Chlamydomonas* constituting a link between circadian rythms and metabolism.

Five MYB TFs are present in this group. Some of them such as *Cre14.g633789* and *Cre03.g198800* are putative orthologs of the *Arabidopsis* genes *At3g27785* and *At5g61620* that have been associated with metabolic regulation [57]. MYB TF factor binding sites have been found significantly enriched in the promoters of genes co-expressed with this group of TFs and TRs. Such as, the triacylglycerol lipase *CrTLL1* (*Cre03.g193500*) and starch phosphorylase *CrPHS1* (*Cre07.g336950*) that present sequences highly similar to MYB binding sites in their promoters (Table 3).

Finally, several genes coding for TFs from the RWP-RK family are members of this group. One of these TFs, *NIT2* (*Cre03.g177700*), has already been shown to be involved in nitrogen and carbohydrate metabolism regulation [58, 59], whereas the other remain to be studied. Promisingly, the RWP-RK TFs *RWP14* (*Cre01.g000050*), *RWP11* (*Cre03.g149400*) and *RWP3* (*Cre14.g612100*) located in this group are putative orthologs of the *Arabdidopsis* genes *RKD5* (*At4g35590*) and *RKD3* (*At5g66990*) that have been shown to be involved in nitrogen and light response [60, 61].

Not surprisingly, TFs in this group seem to constitute an intrincate gene regulatory system with mutual regulations among them. For example, bHLH binding sites can be identified in the promoters of the B-box TF *CrBbox1* (*Cre03.g182700*), the bZIP TF *CrHY5* (*Cre12.g510200*), the bHLH TF *Cre01.g011150* and the MYB TFs *Cre03.g198800* and *Cre14.g621050*. In turn, G-boxes have been found in the promoters of the bHLH genes *Cre01.g011150* and *Cre14.g620850* and the bZIP gene *Cre10.g454850*. Additionally, these TFs seem to exert their regulation in a coordinated manner over the same set of genes since both bHLH and MYB binding sites have been identified in the promoters of genes such as the nitrate transporter *NRT2.3* (*Cre09.g396000*) and the nitrate reductase *NIT1* (*Cre09.g410950*). Such complex interactions are also common in *Arabidopsis*.

#### Autophagy regulation, group b

The TFs and TRs in this group are located in the cluster 1 (orange) identified with green squares in Fig. 6. A GO term analysis of the genes directly linked to them reveals a potential regulation over processes involved in vesicle mediated transport, catabolic process, proteolysis and autophagy. In this group we can find the C3H zinc finger TF *g8693* presenting a high sequence similarity with the *INOSITOL-REQUIRING ENZYME-1b* gene (*At5g24360*) from *Arabidopsis*. This gene is involved in the regulation of the degradation of the endoplasmic reticulum by autophagy [62]. Directly linked to this gene we can find genes involved in autophagy such as *autophagy 9 ATG9* (*Cre09.g391500*) and proteolysis such as *signal peptide peptidase-like 2* (*g18126*). The GATA transcription factor *Cre10.g435450* is also a member of this group and its putative ortholog in *Arabidopsis*, *BME3* (*At3g54810*), has been shown to be involved in response to salt stress [63]. The MYB transcription factor *Cre16.g695600* is also a member of this group whose *Arabidopsis* putative ortholog *At5g06110*, is a heat shock protein involved in stress response [64]. Two genes from the chromatin remodeling family SNF2, *Cre06.g287950* and *Cre06.g270850*, are putative orthologs of *ATRX* (*At1g08600*) and *CHR8* (*At2g18760*), involved in DNA damage response and recombination [65]. The induction of autophagy as a response to diverse stresses has been shown in *Chlamydomonas* [66]. *Cre03.g173165*, *Cre03.g174150* and *g5052* are transcriptional regulators from the BTB/POZ family that are putative orthologs of *ARIA* (*At5g19330*) involved in cellular macromolecule catabolic process [67].

In fact, the OBP binding site was found to be significantly present in the promoters of the genes directly linked to the TFs and TRs in this group (Table 3). This binding site has been shown to be present in promoters of genes induced by oxidative stress in *Arabidopsis* [68]. This is in agreement with the reported autophagy induction by oxidative stress in *Chlamydomonas* [66]. Genes related to autophagy such as *ATG8* (*Cre16.g689650*) and *ATG9* (*Cre09.g391500*) present the OBP binding site in their promoters. Genes involved in vesicle traficking such as Component of the Exocyst Complex *SEC8* (*Cre01.g003050*) and Subunit f the ESCRT-I complex *VPS28* (*Cre16.g678100*) also present the OBP binding site in their promoters (Table 3).

#### Cell cycle regulation, group c

The TFs and TRs of this group are included in the cluster 2 (brown) identified in the previous section as involved in DNA replication, chromosome organization and regulation of cell cycle. These TFs and TRs are highlighted using yellow triangles in Fig. 6. A GO term enrichment analysis over the genes directly linked to these TFs and TRs confirmed their potential regulation over these processes. In this group we can find a MYB3R TF *Cre12.g522400* whose putative orthologs, based on their sequence similarity, are *At5g11510* and *At4g32730* in *Arabidopsis* and *NtmybA1* and *NtmybA2* in *Nicotiana tabacum*. These genes are involved in the G2/M transition during the cell cycle [69–71]. The single member of the YABBY family in *Chlamydomonas* that presents two high mobility group boxes, *Cre16.g672300*, belongs to this group. Its putative ortholog gene *At4g11080* in *Arabidopsis* interacts with mitotic and meiotic chromosomes [72]. Another gene, *ORC1* (*g11180*) belonging to the PHD TF family is also a member of this group. Its putative *Arabidopsis* ortholog *At4g14700* (*Origin recognition complex*) has been shown to be in the core cell cycle machinery involved in the G1/S transition [73, 74]. Several TRs potentially involved in chromatin remodeling are present in this group such as *Cre03.g197700* that code for SET domain containing protein that exhibits a high sequence similarity with *At1g05830* a trithorax protein in *Arabidopsis* [75]. The rest of TFs in this group, *Cre09.g402350* and *Cre12.g516050*, are putative orthologs of *Arabidopsis* genes that have been shown to be co-transcribed with other core cell cycle regulators and TFs in *Arabidopsis* [76].

The E2F motif [77] was found to be the only known motif significantly enriched in the promoters of the genes directly linked to the TFs and TRs in this group (Table 3). The potential orthologs of the genes that contain in their promoters the E2F motif sequence are involved in the G1/S transition such as subunits of the origin of replication complex *ORC1* (*g11180*) and *ORC4* (*Cre17.g726500*), pre-initiation complex subunit *CDC6* (*Cre06.g292850*), DNA replication initiation factor *CDT1* (*Cre03.g163300*), minichromosome maintenance protein *MCM2* (*Cre07.g338000*) and DNA polymerase alpha *POLA1* (*Cre04.g214350*) (Table 3). The presence of the E2F motif in genes regulating the S phase has been shown previously in *Arabidopsis* [78] and *Nicotiana* [77]. The gene *Cre07.g323000*, putative ortholog of the *Arabidopsis* E2F transcription factor, is not included in this group of TFs and TRs. Nevertheless, it is located in its vecinity, suggesting that it may function as an interface between regulation of cell cycle and other processes as it is the case for its *Arabidopsis* ortholog [74]. The two most significant *de-novo* motifs found in our study presents a high similarity with the octamer and hexamer motifs. The combination of these two motifs has been shown to confer S phase-specific transcriptional activation in plants [79]. Genes containing these motifs include B-type cyclin *CYCB1* (*Cre08.g370400*) and cell division cycle protein *CDC45* (*Cre06.g270250*). This suggests a remarkable conservation of cell cycle regulation in the plant kingdom not only limited to the TFs, TRs and their targets involved in this process but also in the cis-regulatory elements, TFBS, present in their promoters.

### Light-regulated transcription factors and transcriptional regulators in ChlamyNET, a tutorial for ChlamyNET usage

In order to ensure the reproducibility of the results presented in this work and to facilitate further and independent studies over the *Chlamydomonas* transcriptome we have developed a web-based software tool also called ChlamyNET. This tool is based on WiGis, a platform for the visualization of large-scale, highly interactive graphs in a user's web browser [80]. The software tool ChlamyNET is available from the web page http://viridiplantae.ibvf.csic.es/ChlamyNet/. In this section we discuss a case study concerning the *Chlamydomonas* potentially light-regulated TFs and TRs that can be used as a tutorial for the use of ChlamyNET.

**Fig. 7:**
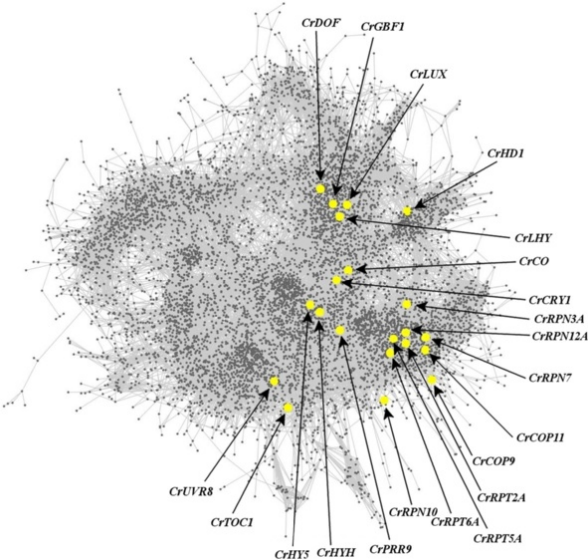
Potentially Light-regulated Transcription Factors and Transcriptional Regulators in ChlamyNET. Twenty-one TFs and TRs exhibiting a high similarity with light regulated TFs and TRs in *Arabidopsis* were identified in ChlamyNET. These genes are not uniformly distributed over ChlamyNET. Clusters 9 (*blue*), 3 (red) and 7 (*green*) were significantly enriched in these potentially ligh-regulated TFs and TRs, so that they are expected to be involved mainly in carbon/nitrogen metabolism, signalling by phosphorylation and protein degradation. The central location of several light-regulated TFs and TRs such as *CrHY5* (*Cre12.510200*) and *CrCRY1* (*Cre06.g295200*) suggests that they are highly authoritative hub genes. Indeed *CrHY5* and *CrCRY1* have 133 and 57 neighbouring genes respectively

Light constitutes the most important source of energy for green algae. Therefore, light is a key environmental signal that affects profoundly *Chlamydomonas* growth and physiology. The study of the light-regulated TFs and TRs co-expression patterns, the biological processes controlled by them and the TFBS where they potentially exert their function can contribute to a better understanding of the response to this key environmental signal in *Chlamydomonas*. Previously, potential orthologs of the light-regulated TFs and TRs in *Arabidopsis* [81] have been identified in the *Chlamydomonas* genome [48]. Most of these TFs and TRs can be found in ChlamyNET. Suprisingly, these genes are not randomly distributed over ChlamyNET (Fig. 7). They are mainly located in clusters 9 (blue), 3 (red) and 7 (green) suggesting that they are specifically involved in carbon/nitrogen metabolism, signalling by phosphorylation and protein degradation. In order to identify genes in the network, the *Search* panel on the left of ChlamyNET can be used. Once the genes are found, they can be selected and information related to their name, putative *Arabidopsis* ortholog, topological indexes, protein domains (Pfam annotation) and neighbours is depicted on the *Selected Gene Details* panel. This information for light-regulated TFs and TRs in ChlamyNET is collected into Table 5.

**Table 5:** Potentially Light Regulated TFs and TRs in ChlamyNET. Their potential *Arabidopsis* ortholog and topological indexes are indicated as well

Chlamydomonas gene	Putative Arabidopsis Ortholog	Number of neighbours	Normalized hub score	Clustering coefficient
Cre06.g295200 CPH1 / CrCRYl	At4g08920 CRYPTOCHROME 1	57	8.12 x 10-^5^	0.39
Cre01.g043150 CrGBF1	At4g36730 G-BOX BINDING FACTOR 1	182	7.36 x 10-^7^	0.31
Cre12.g510200 CrHY5	At5g11260 ELONGATED HYPOCOTYL 5	133	0.32	0.47
Cre06.g310500 CrHYH	At3g17609 HY5-HOMOLOG	39	0.12	0.71
Cre12.g521150 CrDOF	At5g39660 CYCLING DOF FACTOR 2	27	3.79 x 10-^8^	0.34
g6302 CrCO	At5g15840 CONSTANS	58	2.29 x 10-^4^	0.40
Cre02.g094150 CrPRR9	At2g46790 PSEUDO-RESPONSE REGULATOR 9	1	7.72 x 10-^7^	0
Cre06.g275350 CrLHY	At1g01060 LATE ELONGATED HYPOCOTYL	78	5.86 x 10-^7^	0.38
g1542 CrLUX	At3g46640 LUX	38	2.23 x 10-^7^	0.40
Cre06.g277350 CrHD1	At4g38130 HISTONE DEACETYLASE 1	1	5.12 x 10-^18^	0
g16739 CrTOC1	At5g61380 TIMING OF CAB EXPRESSION 1	3	1.49 x 10-^5^	0.33
Cre14.g617350 CrUVR8	At5g63860 UVB-RESISTANCE 8	1	4.09 x 10-^14^	0
Cre05.g234300 CrCOP11	At3g61140 CONSTITUTIVE PHOTOMORPHOGENIC 11	36	7.81 x 10-^14^	0.37
Cre14.g608850 CrCOP9	At4g14110 CONSTITUTIVE PHOTOMORPHOGENIC 9	11	1.26 x 10-^12^	0.27
Cre17.g708300 CrRPN12A	At1g64520 REGULATORY PARTICLE NON-ATPASE 12A	57	7.44 x 10-^14^	0.39
Cre10.g439150 CrRPT5A	At3g05530 REGULATORY PARTICLE TRIPLE-A ATPASE 5A	51	7.49 x 10-^14^	0.45
Cre13.g581450 CrRPN7	At4g24820 REGULATORY PARTICLE NON-ATPASE 7	59	1.52 x 10-^13^	0.28
Cre06.g275650 CrRPN3A	At1g20200 REGULATORY PARTICLE NON-ATPASE 3A	14	2.80 x 10-^15^	0.41
Cre16.g663500 CrRPN10	At4g38630 REGULATORY PARTICLE NON-ATPASE 10	4	3.48 x 10-^14^	0
Cre04.g216600 CrRPT6A	At5g19990 REGULATORY PARTICLE TRIPLE-A ATPASE 6A	19	1.74 x 10-^12^	0.25
Cre07.g329700 CrRPT2A	At4g29040 REGULATORY PARTICLE TRIPLE-A ATPASE 2A	3	3.44 x 10-^16^	0.33

According to this information several light-regulated TFs are highly authoritative hub genes in ChlamyNET such as *CrGBF1* (*Cre01.043150*) and *CrHY5* (*Cre12.510200*) that are co-expressed with more than 130 genes. These genes are involved in photomorphogenesis in *Arabidopsis*, yet their function in *Chlamydomonas* is unknown. Others light-regulated TFs and TRs that constitute hub genes that are co-expressed with more than 50 genes, are *CrCRY1* (*Cre06.g295200*), *CrCO* (*g6302*), *CrLHY* (*Cre06.g275350*) and the different subunits of the 26S proteasome *CrRPN12A* (*Cre17.g708300*), *CrRPT5A* (*Cre10.g439150*) and *CrRPN7* (*Cre13.g581450*). *CrCRY1*, also known as *CPH1*, codes for a putative ortholog of CRY1 in *Arabidopsis* and it is a well known photoreceptor that responds to light stimulus [82]. On the other hand, *CrCO* expression is affected by photoperiod and regulates carbon metabolism and cell cycle progression [2]. Silencing and over-expression of these genes have been shown to massively disrupt *Chlamydomonas* cell growth and proliferation supporting their function as hubs in the network [2]. The potential role of *CrLHY* in circadian rythms and the proteolytic function of *CrRPN12A*, *CrRPT5A* and *CrRPN7* are yet to be tested experimentally. The potentially light-regulated genes *CrHYH* (*Cre06.g310500*), *CrDOF* (*Cre12.g521150*), *CrLUX* (*g1542*) and *CrCOP11* (*Cre05.g234300*) whose putative *Arabidopsis* orthologs are involved in photomorphogenesis, photoperiod response, circardian rythms and protein degradation respectively are co-expressed with around 30 other genes. Recently, *CrDOF* expression has been shown to be influenced by circadian rythms and the photoperiod whereas it directly regulates the expression of *CrCO* [46]. The rest of potentially light-regulated TFs and TRs identified in ChlamyNET are co-expressed with fewer than 20 other genes and are not considered hubs in the network. Most of these genes exhibit a high clustering coeficient in ChlamyNET suggesting a high level of coordination among their co-expressed genes.

**Fig. 8:**
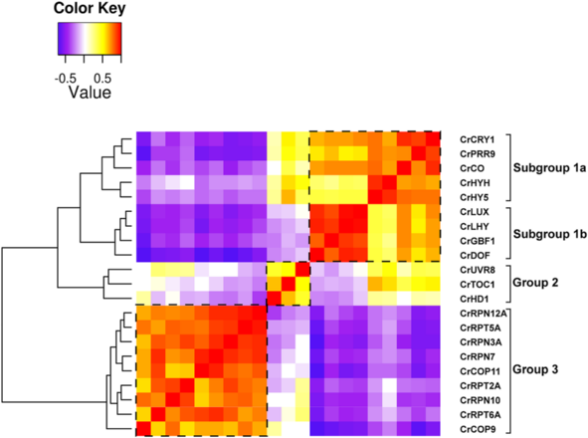
Heatmap Representing the Co-expression Patterns among the Potentially Light-regulated TFs and TRs in ChlamyNET. High positive correlation between the corresponding gene profiles is represented by red/yellow colours, low negative correlation is represented by blue/purple colours. Three different groups are apparent. The first group can be divided into two subgroups. We can observe negative correlations between genes in the subgroup 1b and genes in the second group such as between *CrLHY* and *CrTOC1* which indicates that these two genes may be true orthologs of the circadian clock *Arabidopsis* genes *LHY/CCA1* and *TOC1*. Very low negative correlations are observed between genes in Group 1 and genes in Group 3. Genes coding for different 26S proteasome and signalosome subunits such as *CrRPN7* and *CrCOP9* can be found in. *Group 3*. Their putative *Arabidopsis* orthologs have been described to degrade proteins involved in light response that exhibit a high sequence similarity with those coded by genes in Group 1

In order to study co-expression patterns among a set of selected genes we can generate heatmaps using ChlamyNET. In this type of graphs we represent the correlation among expression profiles of a selected set of genes. Heatmaps can be generated in ChlamyNET using the *Analysis* section located in the *Search* panel once a set of genes have been selected. Additionally, genes at distance one, two or three from the selected genes can be included in the heatmap. As an example, a heatmap depicting the co-expression patterns among the potentially light-regulated TFs and TRs previously selected is presented in Fig. 8. We can observe three different groups. Genes in the same group exhibit a high positive correlation (red/yellow colours) whereas genes from different groups present a very low negative correlation (blue/purple colours). In group 1 we can distinguish two subgroups. On the one hand, in subgroup 1*a* it can be observed that *CrCRY1*, *CrPRR9*, *CrCO*, *CrHY5* and *CrHYH* are highly co-expressed. This could indicate co-regulation of these genes by the same factors or regulation among themselves. On the other hand, in subgroup 1*b* we find that *CrLUX*, *CrLHY*, *CrGBF1* and *CrDOF* have a very high co-expression value. These two subgroups are also co-expressed and hence form a single group. Group 2 is composed of three genes, *CrUVR8*, *CrTOC1* and *CrHD1*. This group of genes presents a negative correlation with genes in subgroup 1*b* which makes them constitute a separate group. For instance, *CrLHY* and *CrTOC1* are negatively co-expressed as expected if they are true orthologs of the *Arabidopsis* circadian genes *LHY/CCA1* and *TOC1*. These genes form a negative feedback loop that constitutes the core of the circadian clock in *Arabdiopsis*. Group 3 exhibits a very low negative correlation with group 1 and a moderate negative correlation with group 2. The genes in this group are different members of the 26S proteasome and signalosome subunits such as *CrRPN7* and *CrCOP9*. Their putative *Arabidopsis* orthologs have been described to degrade proteins involved in light response [61]. This suggests an antagonist expression pattern between light-regulated TFs and different subunits of the 26S proteasome and signalosome that is already established in *Chlamydomonas*. This would need further research and experimental validation, but can be an important clue for a preliminary investigation.

The biological processes potentially controlled by the light-regulated TFs and TRs in ChlamyNET can be deduced by applying GO terms enrichment over their co-expressed genes. This can be performed by using the *Analysis* section located in the *Search* panel once the neighbouring genes have been selected. As described in the Methods section we can combine the significative GO term identified, based on orthology, with those determined based on conserved protein domains (Additional file 6: Table S2). According to this methodology, the potentially light-regulated TFs and TRs are co-expressed with genes involved in ion transport, for example the nitrate transporter *NRT2.2* (*Cre09.g410800*) and Mo-molybdopterin cofactor biosynthesis such as *MoeA* (*Cre10.g451400*), which produces essential cofactors for the nitrate reductase, a key enzyme in the nitrate metabolism. Additionally, carbohydrate metabolism appears as a significative GO term, including genes involved in starch and glucose degradation such as the starch phosphorylase *CrPHS2* (*Cre12.g552200*), the alpha-amylase *AMY* (*Cre08.g384750*) and the glucose-6-phosphate dehydrogenase *GLD2* (*Cre08.g378150*). Finally, protein phosphorylation is another relevant significative GO term with genes potentially involved in cell cycle control such as the mitogen-activated protein kinase kinase kinases *CrMKKK1* (*Cre07.g347000*) and *CrMKKK2* (*Cre02.g108650*). Therefore, this analysis suggests a potential regulation of carbon/nitrogen metabolism and cell cycle through protein phosphorylation by these potentially light-regulated TFs and TRs that needs to be experimentally validated.

Finally, selecting the switch *Promoter Sequence Enrichment* in the *Search* panel, a transcriptional factor binding site (TFBS) enrichment analysis over the promoters of selected genes can be performed . In this case, several significative light-regulated TFBS in *Arabidopsis* such as SORLIP2, SORLIP3 and SORLREP5 [83] were identified. For example, the *CrGBF1*, *CrHYH* and *CrLUX* genes present the sequence SORLIP3 in their promoters. This is in agreement with their high co-expression values (Fig. 8). The presence of these TFBS in the promoters of the genes studied here suggests a high conservation of light regulated TFBS across the green lineage.

A more detailed presentation of this case study is available from the web page of ChlamyNET, http://viridiplantae.ibvf.csic.es/ChlamyNet/.

### Experimental validation

ChlamyNET aims at becoming an enabling technology for researchers on the *Chlamydomonas* transcriptome. For example, ChlamyNET can be used to predict changes in gene expression. When a specific gene is mutated or overexpressed, ChlamyNET predicts that the expression of genes located in the neighbourhood of the mutated or overexpressed gene will be affected, whereas genes in distant regions will not substantially change their expression profile. Specifically, another application of ChlamyNET is predicting targets of specific TFs. TFs and their targets tend to be strongly co-expressed since TFs directly regulate the expression of their target genes. Therefore, the targets of a TF should be contained in its neighbourhood, possibly directly linked to it. Additionally, when the sequences recognised by a TF are well characterized, the identification of these sequences in the promoters of genes co-expressed with it provides more convincing evidence for these genes being direct targets of the corresponding TF. In this way, the analysis of gene neighbourhoods and the significance of TFBS in gene promoters can be studied using ChlamyNET constituting a powerful tool for gene expression analysis.

**Fig. 9:**
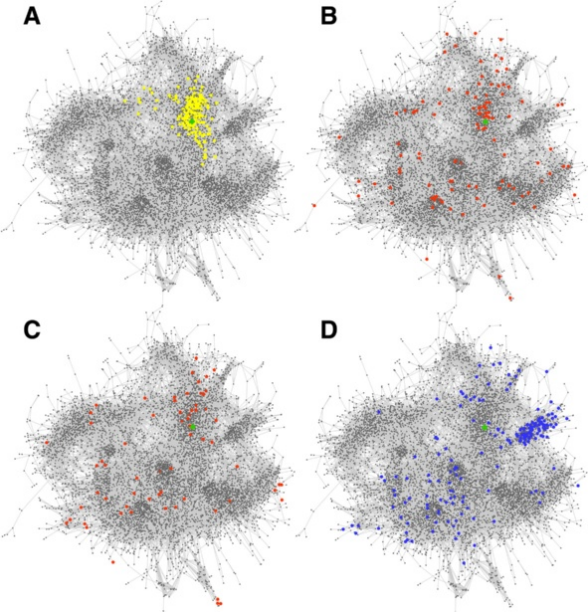
Experimental Cross-validation of the Predictive Power of ChlamyNET using RNA-seq Data from Algae Overexpressing the *CrDOF* gene. **a** The *CrDOF* gene (identified as a green diamond in ChlamyNET) has a neighbourhood at distance two consisting of 216 genes represented in yellow. These genes showed an average fold-change increase of 2*.*7 which is significantly higher than the fold-change in the rest of ChlamyNET with a p-value of 5*.*63° 10 ^−3^. **b** Genes increasing their expression level in LD conditions at least by a four fold-change in the CrDOF genotype when compared to the wild type CW15 are represented in red. Note that the neighbourhood of the *CrDOF* gene, represented in green, is enriched in these genes according to a p-value of 0*.*029 obtained using Fisher's exact test. **c** Genes increasing their expression level in SD conditions at least by a four fold-change in the CrDOF genotype when compared to the wild type CW15 are represented in red. These genes tend to group around the *CrDOF* gene, represented in green. **d** Inhibited genes in LD conditions in the *CrDOF*in genotype when compared to the wild type CW15 with at least by a four fold-change are represented in blue. Note that cluster 2 (brown) involved in DNA replication and cell cycle processes is significantly enriched in these genes

In this section, we present an experimental validation of the predictive power of ChlamyNET based on one of the most widely used methodologies, cross-validation. In this methodology, once a predictor has been constructed, an independent data set is used to evaluate its performance. As it is common in the study of *Chlamydomonas*, the data used to construct ChlamyNET was obtained from algae grown on continuous light (LL) conditions without a dark period. In spite of this, probably due to the fact that the different experiments were carried out with different light intensities, we were able to identify patterns of co-expression between TFs and TRs that potentially respond to light stimuli as discussed in the previous section. Additionally, none of the genotypes used in the construction of ChlamyNET consisted of an overexpressor of a gene involved in photoperiod response. Therefore, in order to use a totally independent data set from those used to construct ChlamyNET we generated RNA-seq data from algae grown in long day (LD) conditions (16 hours of light and 8 hours of dark) and in short day (SD) conditions (8 hours of light and 16 hours of dark). Two different genotypes were analyzed. As wild type we used the *Chlamydomonas* strain CW15 and for a strain affecting the response to photoperiod we chose algae that overespressed the *CrDOF* gene under a nitrate inducible promoter. As discussed in the previous section the gene *CrDOF* (*Cre12.g521150*) is a potentially light-regulated transcription factor that has recently been shown to response to photoperiod and circadian rythms [46]. This last *Chlamydomonas* strain was called *CrDOF*in. For more details on algae material, growth conditions and RNA-seq see the Methods section. Using the RNA-seq data analysis protocol described in the Methods section we determined the gene expression level fold-change when comparing *CrDOF*in to CW15 (Fig. 9). According to the predictions provided by ChlamyNET the 216 genes in the neighbourhood at distance two from the *CrDOF* gene, yellow dots in Fig. 9a, are expected to increase their expression. Indeed, 69*.*44 % of these *CrDOF* neighbouring genes increased their expression level in the *CrDOF*in strain when compared to the wild type strain CW15 in LD conditions. Actually, the *CrDOF* neighbouring genes showed an average fold-change increase of 2*.*7 which is significantly higher than the fold-change in the rest of ChlamyNET with a p-value of 5*.*63°10^−3^. Additionally, we identified in ChlamyNET those genes that increased their expression level a fold-change of four in the genotype *CrDOF*in when compared to the wild type CW15 in LD and SD conditions (Fig. 9b and c). The neighbourhood of the gene *CrDOF* at distance two was shown to be significantly enriched with a p-value of 0*.*029 in genes that increase their expression level a fold-change of 4 in LD conditions. This further supports the predictive power of ChlamyNET in LD conditons (Fig. 9b). Nevertheless, the highly activated genes in SD conditions appeared scattered all over ChlamyNET without concentrating in the *CrDOF* neighbourhood at distance two (Fig. 9c). This shows a limitation in the predictive power of ChlamyNET. It should be recalled that all data collected for the construction of ChlamyNET correspond to LL conditions. The data from LD conditions is somehow similar to these data and therefore, ChlamyNET was a good predictor for this condition. On the other hand, SD conditions, in which the dark period is longer than the light period, represent very different conditions from those used for the data with which ChlamyNET was generated. Therefore, ChlamyNET performs poorly as a predictor for this condition.

We also identified in ChlamyNET those genes that showed a 4-fold decrease in expression level in *CrDOF*in compared to the wild type CW15 in LD conditions (Fig. 9d). These genes were not located in the neighbourhood of *CrDOF*, suggesting that it acts as a direct activator and, possibly, as an indirect repressor in LD conditions. Instead, cluster 2 (brown) was significantly enriched with these highly inhibited genes with a p-value of 2*.*2°10^−16^. This provides evidence about *CrDOF* being involved in cell-cycle regulation, which indeed was experimentally validated [46].

**Fig. 10:**
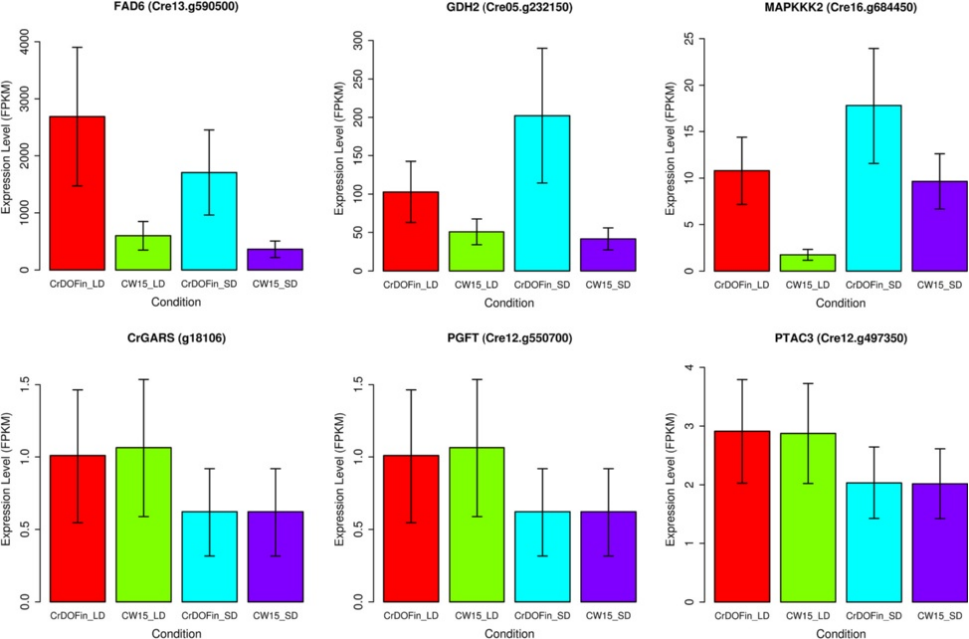
Expression Levels (FPKM) of several *CrDOF* Neighbouring Genes and Distant Genes in the *CrDOF*in and CW15 Genotypes Grown in LD and SD Conditions. Three genes in the neighbourhood at distance two from *CrDOF* were chosen to illustrate the correct prediction provided by ChlamyNET with respect to their increase in expression level in the *CrDOF*in genotype when compared to CW15. These genes are *fatty acid desaturase FAD6* (*Cre13.g590500*) involved in carbon metabolism, *glutamate dehydrogenase GDH2* (*Cre05.g232150*) involved in nitrogen metabolism and *serine/threonine kinase MAPKKK2* (*Cre16.g684450*) possibly involved in cell cycle regulation. Additionally, we selected three genes from the purple cluster located far away from the *CrDOF* gene in ChlamyNET to show that distant genes expression is not substantially affected by *CrDOF* overexpression. These genes are *glycinamide ribonucleotide synthetase CrGARS* (*g18106*), *phosphoribosylglycinamide formyltransferase PGFT* (*Cre12.g550700*), both involved in purine biosynthesis and *plastid transcription factor PTAC3* (*Cre12.g497350*) involved in regulation of plastid genes

In order to illustrate the effect of the overexpression of *CrDOF* over its neighbouring genes we chose three genes at distance two from it and represented their expression level in the four conditions, *CrDOF*in and CW15 grown in LD and SD. We chose three genes that represent the main biological processes affected by the potentially light-regulated TFs and TRs according to the results from the previous section. These genes are a fatty acid desaturase *FAD6* (*Cre13.g590500*) involved in carbon metabolism, a glutamate dehydrogenase *GDH2* (*Cre05.g232150*) involved in nitrogen metabolism and a serine/threonine kinase *MAPKKK2* (*Cre16.g684450*) possibly involved in cell cycle regulation (Fig. 10). As expected, these genes increased their expression levels in the *CrDOF*in genotype when compared to CW15. Additionally, we chose three genes located in the cluster 4 (purple), far away from *CrDOF*, to show that the expression level of distant genes tend to remain unchanged. These genes are a glycinamide ribonucleotide synthetase *CrGARS* (*g18106*), a phosphoribosylglycinamide formyltransferase *PGFT* (*Cre12.g550700*), both involved in purine biosynthesis and a plastid TF *PTAC3* (*Cre12.g497350*) involved in regulation of plastid genes. All these experimental tests validated the predictive power of ChlamyNET.

## Conclusions

This gene co-expression network and its associated web-based tool ChlamyNET constitute one of the first integrative approaches to the study of the *Chlamydomonas* transcriptome. They aim at providing researchers with an enabling technology that will allow them to study gene co-expression patterns, determine significant biological processes, molecular functions and cellular components for a set of genes of interest as well as to identify significant TFBS in the promoters of a given set of genes.

In this work, we have shown that ChlamyNET exhibits non-random topological properties, namely scale-free and small-world properties. This suggests that the *Chlamydomonas* transcriptome posseses relevant characteristics related to error tolerance, vulnerability and information propagation [28]. On theone hand, the scale-free property implies robustness against random gene mutations or error tolerance, which means that since most genes are only co-expressed with a few other genes, a random mutation is likely to affect a non important gene altering the expression of a reduced number of other genes.Nevertheless, the existence of key authoritative hub genes produces fragility or vulnerability to targeted attacks against this type of genes. The removal or mutation of an authoritative hub gene would affect a large number of other genes co-expressed with it, massively disrupting the functioning of the *Chlamydomonas* transcriptome. This can lead to lethality or defective growth. For example, this has been shown for the authoritative hub gene *CrCO* (*g6302*) whose over-expression and silencing are detrimental for cellular growth [2]. Additionally, the small-world property facilitates a quick spreading of information throughout ChlamyNET.

The analysis of the location of hub genes and genes with high clustering coefficient shows that both of them group together in specific regions of ChlamyNET. This indicates the existence of gene clusters whose expressions are highly coordinated, possibly to perform related biological processes. Indeed, we identified nine gene clusters that present a high intra-cluster and a low inter-cluster co-expression. Among these clusters we highlight the results obtained in two of them, clusters 1 and 2.

The most central cluster (blue cluster), where most authoritative hub genes are located, is significantly enriched in genes involved in carbon/nitrogen metabolism, signalling through protein phosphorylation and light response. This cluster is also significantly enriched in TFs, revealing a high transcriptional control over carbon/nitrogen metabolism induced by light. Several bHLH TFs are contained in this cluster and only one of them, *CrbHLH1* (*Cre14.g620850*), presents a potential *Arabidopsis* ortholog, *PAR1* (*At1g69010*), which has been shown to be involved in light response, and indeed the E-box sequence, a bHLH binding site, was found to be significantly present in the promoters of genes in this cluster. Two TFs from the bZIP family are also located in this cluster, *CrHY5* (*Cre12.g510200*) and *CrHYH* (*Cre06.g310500*), potential orthologs of the *Arabidopsis HY5* (*At5g11260*) and *HYH* (*At3g17609*) genes, respectively. Again, the G-box sequence, a TFBS recognized by *HY5* and *HYH* in *Arabidopsis*, was found to be significantly present in the promoters of their co-expressed genes. Additionally, this cluster contains two ARR-B TFs (*Cre13.g572450* and *g16739*) and the B-box TF *CrBbox1* (*Cre03.g182700*) whose potential *Arabidopsis* orthologs *RR14* (*At2g01760*), *TOC1* (*At5g61380*) and *COL1* (*At5g15850*) are involved in circadian rythms. This suggests a key role played by light in the regulation of central metabolism in*Chlamydomonas* mediated by TFs from the bHLH and bZIP families. It also provides evidence for an input from circadian rythms exerted by genes from the ARR-B and B-box families.

One of the most peripheral clusters in ChlamyNET, cluster 2 (brown), was shown to be involved in DNA replication and transitions between the cell cycle phases. This cluster contains potential orthologs of *Arabidopsis* genes involved in the G1/S transition such as origin of replication complex *ORC1* (*g11180*) and *ORC4* (*Cre17.g726500*), pre-initiation complex subunit *CDC6* (*Cre06.g292850*), DNA replication initiation factor *CDT1* (*Cre03.g163300*), minichromosome maintenance protein *MCM2* (*Cre07.g338000*) and DNA polymerase alpha *POLA1* (*Cre04.g214350*). The E2F sequence was found to be significantly present in the promoters of these genes. Additionally, the presence of a combination of the octamer and hexamer motifs was significantly enriched in the promoters of the genes from this cluster such as the B-type cyclin *CYCB1* (*Cre08.g370400*). The E2F sequence and the combination of octamer and hexamer motifs have been shown to confer S phase-specific transcriptional activation in higher plants.

These results suggest that key elements in the regulation of cell cycle, light response and carbon/nitrogen metabolism are already established in *Chlamydomonas* and conserved in higher plants such as *Arabidospsis*. The conserved elements are not only limited to TFs, TRs and their targets but also include the *cis*-regulatory elements, TFBS, present in their promoters.

The web-based software tool ChlamyNET (http://viridiplantae.ibvf.csic.es/ChlamyNet/) was developed to ensure the reproducibility of the results presented in this work and to facilitate further and independent studies over the *Chlamydomonas* transcriptome. We used potentially light-regulated TFs and TRs to illustrate its functionalities. Our case study suggests that these genes regulate carbon/nitrogen metabolism and cell cycle. Additionally, light regulated TFBS in *Arabidopsis* such as SORLIP2, SORLIP3 and SORLREP5 were identified in their gene promoters. Several other cases studies are available from the web page of ChlamyNET, http://viridiplantae.ibvf.csic.es/ChlamyNet/.

Finally, RNA-seq data from algae overexpressing the transcription factor *CrDOF* involved in photoperiod response were used as an independent data set to experimentally cross-validate the predictive power of ChlamyNET.

## Methods

### Data acquisition and processing

In this study we used RNA-seq data from the *Chlamydomonas* transcriptome publicly available at the *Sequence Read Archive* (SRA, http://www.ncbi.nlm.nih.gov/Traces/sra/) [84], a database resource at the *National Center for Biotechnology Information* (NCBI) that stores more than 500 TeraBases of next-generation sequencing data. We collected more than 287 GigaBytes of information produced by seven different studies [10–16] consisting of 50 samples representing eight different genotypes under diverse physiological conditions, see Additional file 1: Table S1. These data provide a general overview of the *Chlamydomonas* transcriptome in a physiologically relevant context. All these data were obtained using the same next-generation sequencing platform, Illumina Genome Analyzer [85], in order to facilitate the comparison between samples from different experiments.

The available *Chlamydomonas* sequenced genome (version 5.3) [1] was downloaded from Phytozome (http://www.phytozome.net/) [86], a web-based platform for green plant genomics, in order to be used as the reference genome in our study. Additionally, we also obtained from the same web resource the corresponding Augustus u11.6 gene annotation that was used as a reference transcriptome.

The processing of raw sequencing data when a reference genome is available can be divided into three different stages: (i) filtering out low quality reads and alignment of reads to the reference genome; (ii) assembly of transcripts; and (iii) estimation of gene expression [87]. In our study, we followed the methodology described in [88] that makes use of the free software packages Tophat [89] and Cufflinks [90]. First, we perfomed the preprocessing of the raw data consisting of the fastq files from each sample. The read sequences of low quality were filtered out according to their Phred quality scores [91, 92] and the remaining ones were aligned to the reference genome with the software package Tophat that in turn makes use of the fast and memory efficient short read aligner Bowtie [93]. Most of the analyzed samples were of good quality and produced a high alignment rate greater that 80 %. The alignments of read sequences to the reference genome produced in this step were stored in BAM (binary aligment maps) files.

In the second step, we used the alignments in the BAM files and the known transcripts from the Augustus u11.6 annotation for the assembly of the sample specific transcriptomes using the software package Cufflinks. The whole transcriptome identified in all the samples was integrated and stored in a GTF (gene transfer format) file using Cuffmerge, a utility program within the Cufflinks package. We performed this refinement of the currently available annoted *Chlamydomonas* transcriptome in order to avoid incomplete or incorrect annotation that could reduce accuracy [90] in our study.

Finally, the gene expression levels in the different conditions integrated in our study were estimated using Cuffdiff, a program included in the Cufflinks package. In order to avoid biases due to transcript length and the total number of reads generated in each experiment, we used as unit of measurement *fragments per kilobase of transcript per million mapped fragments* (FPKM) [90, 94]. Additionally, recent suggestions for normalization methods [95] that reduce the bias due to the non-uniform distribution of mapped reads within transcripts were taken into account by setting the corresponding parameters in Cuffdiff. These normalizations remove the biases in the data while preserving the variation in gene expression that occurs because of biologically relevant changes in transcription, allowing the comparison of gene expression across multiple experiments. Subsequent analysis and visualization of the results were performed using the R package cummeRbund [96].

### Selection of di erentially expressed genes

The selection of differentially expressed genes was performed using the standard methodology applied to the analysis of RNA-seq data described in [97]. The logarithm of the levels of expression measured in FPKM were computed and the delta method to estimate the variance of the log odds was used. Those genes that exhibited an adjusted p-value for the multiple testing lower than 0*.*05 were considered to be differentially expressed.

### Gene Co-expression criterion and network construction

We used the absolute value of the *Pearson correlation coefficient* between gene expression profiles across the different conditions to determine the level of co-expression between the selected genes [30]. For each possible correlation value *cor*, we represented the co-expression relationships between genes that exceed this value in the *Chlamydomonas* transcriptome using an undirected weighted network *G*_*cor*_ = (*V*_*cor*_*,E*_*cor*_). The nodes or vertices in *V*_*cor*_ correspond to the genes. An undirected edge (*g*_1,_*g*_2_) in *E*_*cor*_ with associated weight *w > cor* indicates the existence of a significant co-expression relationship between genes *g*_1_ and *g*_2_ with an absolute value of the *Pearson correlation coefficient* between the corresponding expression profiles equal to *w*.

In order to decide which correlation value is high enough to consider that two genes are significantly co-expressed we used a criterion that establishes a compromise between the generation of a scale-free network and a high network density. Most biological networks characterized so far are scale-free, which makes this property the most common metric for the rational selection of a gene correlation threshold. In order to facilitate the detection of clusters or modules of genes in the constructed network we also added the restriction of generating a network with a high density [25].

A range of correlation thresholds were considered. For each possible correlation cut-off value we determined how close the corresponding network was to fullfil the scale-free condition by computing the *R*^2^ of the linear regression for the logarithmic transform of the node degree distribution. Additionally, for each possible cut-off value we used the average node degree as a measurement of the network density.

### Network visualization

Graphical representations of the network were perfomed using Cytoscape [98], a software package for network visualization and data integration. Specifically, the organic layout method was applied to visualize ChlamyNET. This algorithm consists of a variant of the force-directed layout. Nodes produce repulsive forces whereas edges induce attractive forces. Nodes are then placed such that the sum of these forces are minimised. The organic layout has the effect of exposing the clustering structure of a network. In particular, this layout tends to locate tighly connected nodes with many interactions or *hub nodes* together in central areas of the network.

### Significance of topological properties

In order to determine the statistical significance of the scale-free property of ChlamyNET we generated 10^4^ random networks with the same number of nodes and edges as ChlamyNET following the *Erdös and Renyi random graph model* [99]. None of these random networks exhibited a scale-free topology similar to ChlamyNET. This indicates that the scale-free topology of ChlamyNET is not random but rather it is the product of a self-organizing process. It has been suggested that scale-free networks emerge from a growth process by which newly added nodes preferentially attach to already existing nodes with a high number of neighbours [27]. In the case of ChlamyNET the scale-free feature can be a consequence of two mechanisms in the evolution of gene co-expression networks: (i) gene co-expression networks are not static, instead new genes may appear; and (ii) new genes are preferentially co-expressed with genes that already exhibit a large number of co-expressed genes.

We also studied the clustering coefficient in ChlamyNET, a measurement of the density of edges or co-expression relationships around genes. The clustering coefficient of a gene is calculated as the ratio of the actual number of co-expression relationships among all its neighbours and the maximal possible number of such co-expression relationships.

In order to determine that the global clustering coefficient of ChlamyNET is significantly high we generated 10^4^ random scale-free networks with the same number of nodes and edges as ChlamyNET following the *Barabasi random scale-free graph model* [27]. None of these random networks exhibited a clustering coefficient higher than ChlamyNET.

### Clustering techniques

In a general way, clustering techniques aim at identifying groups or clusters whose individuals exhibit high similarities, whereas individuals from different groups or clusters present low similarities. When clustering techniques are applied to co-expression networks, the similarity among genes is meassured using the correlation among the corresponding gene profiles or co-expression. Therefore, the goal of clustering techniques, when applied to gene co-expression data, consists on identifying disjoint groups or clusters of genes so that the co-expression between genes in the same cluster is high (intra-cluster similarity) whereas the co-expression between genes from different clusters is low (inter-cluster similarity) [36]. In this respect, the silhouette [36] a criterion that combines the minimization of inter-cluster similarity with the maximization of the intra-cluster similarity, is one of the most popular measurements for the assesment of a clustering analysis. In our study we used this criterion to determine which clustering algorithm and number of clusters best describes the underlying structure in ChlamyNET. We compared the performance of the two most widely used clustering algorithms, hierarchical clustering and partition around medoids (PAM) for different number of clusters ranging from 4 to 20 clusters using the R package *clValid* [36].

### Gene ontology term enrichment analysis

The *Chlamydomonas* transcriptome is very sparsely annotated since experimental validation of the different computationally predicted functions is still missing for most genes. In order to overcome this lack of GO term annotation, we followed two diffeerent complementary approaches. In our first approach, we assigned to each *Chlamydomonas* gene the GO terms associated with its potential *Arabidopsis* ortholog based on sequence similarity. In our second approach, we used the annotation about protein domains and tools available from the Pfam database [35] to determine the protein family to which each *Chlamydomonas* gene belongs to. The GO terms associated to the identified protein family were then assigned to the corresponding gene. Our methodology for the identification of the GO terms over-represented in each cluster is a combination of both approaches. We identified as overrepresented GO terms those found by both approaches or by only one of them with a very high statistical signi cance (a p-value lower than 10 ^−6^). The R package *topGO* was used to perform GO term enrichment using Fisher's exact test. As gene background we selected the entire *Chlamydomonas* gene set as indentified in the Phytozome 9.1 database.

### Transcription factor binding site enrichment analysis

Transcription Factor Binding Sites (TFBS) enrichment analysis was performed using HOMER [100] and the known TFBS sequences in plants from the databases AGRIS [101], JASPAR [102] and AthaMap [103]. The findMotifs.pl script, applying the default parameters, was used to perform a known and de-novo motif over-representation analysis.

The background used for the over-representation analysis consists of all the gene promoters annotated in the current version of the *Chlamydomonas* genome. These data were downloaded using the BioMart functionality associated with Phytozome.

### Alga material, growth conditions and RNA sequencing

Two independent biological replicates of *Chlamydomonas reinhardtii* wild type CW15 [104], and the transgenic line *CrDOF*in [46], were grown in flasks with the induction media Sueoka NO3- [105] under LD (16 h light/8 h dark) or SD (8 h light/16 h dark) conditions at 50 E light intensity with 22 *C* (during light period) and 18 *C* (during night period) in a model SG-1400 phytotron (Radiber SA, Spain). Algal cells were grown during 4 days and then, were harvested 4 hours after the light went on, which was considered at Zeitgebertime zero (ZT0). The RNA isolation was performed by TRIZOL (Invitrogen) method following the manufacturer instruction. RNA quality was tested employing a ND-1000 Spectrophotometer (Nanodrop). Library preparation was carried out following the manufacturer's recommendations. Sequencing of RNA libraries was performed with the Illumina HiSeq 2000 sequencer, yielding approximately 40 million 50 bp long reads for each sample.

### Availability of supporting data

The RNA-seq data set used to cross-validate the preditive power of ChlamyNET is available at European Nucleotide Archive identified with the accession number PRJEB6682.

The processed RNA-seq data, R scripts used in the construction and analysis of our network as well as the network itself in gml format are available from the web page http://viridiplantae.ibvf.csic.es/ChlamyNet/.

## Additional files

Additional file 1: Table S1. Brief description of the data used in this analysis including genotypes, conditions and sequence information. (PDF 19 kb) Additional file 2: Figure S1. Gene Cluster Expression Profiles. The normalized expression of every gene in each cluster is represented in grey. For each cluster the red line represents the mean positive expression profile whereas the blue line represents the mean negative expression profile. Recall that the absolute value of the correlation is used to define co-expression. Note that the expression profiles for each cluster are distinct. (TIFF 7917 kb) Additional file 3: Figure S2. Metabolic Pathways Contained in Clusters 2 (Brown), 3 (Red) and 7 (Green) from Figure 4. Analysis of the *Chlamydomonas* metabolic pathways revealed that cluster 2 (brown) in Figure 4 is enriched in metabolic processes involved in the DNA and RNA synthesis such as the pyrimidine synthesis pathways. Nevertheless, clusters 3 (red) and 7 (green) (Figure 4) were not so significantly enriched in metabolic pathways. Nevertheless, in cluster 3 (red), with a low significance, the synthesis pathway for triacylglycerol using galactolypids produced by glycolipid desaturation as acyl donors, was identified. In cluster 7 (green), the aerobic respiration pathway was fully included. (PNG 50 kb) Additional file 4: Figure S3. Metabolic Pathways Contained in Clusters 9 (Blue) and 8 (Turquoise) from Figure 4. Analysis of the *Chlamydomonas* metabolic pathways revealed that cluster 9 (blue) and 8 (turquoise) (Figure 4) are enriched in diverse metabolic processes. Cluster 9 (blue) contains genes involved in core carbon/nitrogen metabolic pathways such as starch biosynthesis, the oxydative branch of the pentose phosphate pathway and nitrogen assimilation pathways co-expressed with the needed molybdenum cofactor synthetic pathway. Cluster 8 (turquoise), the two most important metabolic pathways in photosynthetic organisms, the Calvin cycle and the TCA cycle, have a significant number of genes represented. (PNG 98 kb) Additional file 5: Figure S4. Metabolic Pathways Contained in Cluster 1 (Orange) and 4 (Purple) from Figure 4. Analysis of the *Chlamydomonas* metabolic pathways revealed that cluster 1 (orange) and 4 (purple) (Figure 4) are enriched in specific metabolic processes. Cluster 4 (purple) comprises metabolic pathways associated with protein synthesis such as the tRNA charging pathways, aminoacid biosynthesis and the non-oxydative branch of the pentose phosphate pathway. The TAG biosynthetic pathway that preferentially uses as acyl donors galactolipids is also a member of this cluster. Cluster 1 (orange) is enriched in genes involved in lipid metabolism, including the TAG biosynthetic pathway which uses phospholipids that are in turn produced in the phosphatidylinositol pathway as acyl donors. The pathway for the synthesis of the necessary coenzyme A, is also present in this cluster. (PNG 119 kb) Additional file 6: Table S2. Biological Processes Potentially Controlled by the Light-regulated TFs and TRs in ChlamyNET (PDF 8 kb)
