# Supplementary material for: ChlamyNET: a Chlamydomonas gene co-expression network reveals global properties of the transcriptome and the early setup of key co-expression patterns in the green lineage
Source: BMC Genomics. 2016 Mar 12;17:227. doi: 10.1186/s12864-016-2564-y (PMC4788957; doi:10.1186/s12864-016-2564-y)
Supplement: Additional file 1: Table S1. — Brief description of the data used in this analysis including genotypes, conditions and sequence information. (PDF 19 kb) [file 12864_2016_2564_MOESM1_ESM.pdf]

Experiment	Condition	Sample	Size	Number of Reads	Read Length
Analysis of the transcriptome from Fe-repleted, Fe-limited and Fe-deprived Chlamydomonas cells grown in TAP and minimal media	WT strain 2137 Fe-repleted TAP	SRR402019	2.1	8.67	70
		SRR402020	2.2	9.38	70
	WT strain 2137 Fe-deficient TAP	SRR402021	2.3	9.85	70
		SRR402022	2.2	9.33	70
		SRR402023	2.2	9.20	70
	WT strain 2137 Fe-limited TAP	SRR402024	2.3	9.63	70
		SRR402025	2.3	9.62	70
		SRR402026	2.0	8.51	70
	WT strain 2137 Fe-repleted Minimal Medium	SRR402027	5.4	11.00	200
		SRR402028	6.0	19.23	100
		SRR402029	7.2	14.64	200
		SRR402030	8.5	27.13	100
	WT strain 2137 Fe-deficient Minimal Medium	SRR402031	6.2	12.70	200
		SRR402032	6.8	21.58	100
		SRR402033	6.7	13.68	200
		SRR402034	7.9	25.32	100
	WT strain 2137 Fe-limited Minimal Medium	SRR402035	4.8	9.87	200
		SRR402036	6.6	21.04	100
		SRR402037	6.7	13.80	200
		SRR402038	8.6	27.60	100
Impact of oxidative stress on the transcriptome	WT strain 2137 t=0 of H_2O_2 treatment	SRR394058	8.0	30.27	76
		SRR394059	39.48	21.04	76
	WT strain 2137 t=0.5 of H_2O_2 treatment	SRR394060	8.8	33.03	76
		SRR394061	8.1	30.54	76
	WT strain 2137 t=1 of H_2O_2 treatment	SRR394062	8.3	31.28	76
		SRR394063	13.0	47.92	76
Singlet oxygen acclimation	WT strain 4A+	SRR363977	0.91	5.4	36
		SRR363978	0.93	5.26	36
	sor1 mutant	SRR363979	2.1	11.24	36
Different TAP recipes	Recipe1	SRR074357	1.3	6.52	35
	Recipe2	SRR074358	1.4	7.00	35
Analysis of the transcriptome from Cu-deficient and Cu-sufficient Chlamydomonas wild type and crr1 mutant cells grown in TAP and minimal media	WT strain 2137 Copper deficient cells grown in TAP medium	SRR096493	0.61	4.38	17
		SRR096494	0.44	3.13	17
		SRR096495	2.00	7.45	70
		SRR096496	1.30	6.70	35
		SRR096497	1.30	6.65	35
		SRR096498	0.75	4.47	33
		SRR096499	0.72	4.26	33
		SRR096500	0.70	3.79	33
		SRR096501	0.92	5.01	33
	WT strain 2137 Copper deficient cells grown in TAP medium	SRR096502	0.60	4.26	17
		SRR096503	0.57	3.80	17
		SRR096504	1.8	6.68	70
		SRR096505	1.3	6.73	35
		SRR096506	1.4	7.03	35
		SRR096507	0.78	4.64	33
		SRR096508	0.80	4.67	33
		SRR096509	0.90	4.88	33
		SRR096510	0.90	4.89	33
	Crr1-2 mutant Cu-deficient cells grown in TAP medium	SRR096511	0.34	2.44	17
		SRR096512	0.68	4.87	17
		SRR096513	1.50	7.43	35
		SRR096514	1.40	6.83	35
	Crr1:crr1 strain Cu-deficient	SRR096515	1.3	6.66	35
		SRR096516	1.4	7.13	35
	WT strain 2137 Cu-deficient Min	SRR096517	3.4	11.49	100
		SRR096518	3.9	13.26	100
	WT strain 2137 Cu-sufficient Min	SRR096519	3.6	12.40	100
		SRR096520	3.6	12.25	100
Global changes following N-deprivation	WT strain cw15 Nitrogen repletion	SRR066643	4.30	16.90	75
		SRR066644	4.70	18.30	75
		SRR066645	4.50	17.81	75
	WT strain cw15 Nitrogen deprivation	SRR066646	4.50	17.66	75
		SRR066647	4.60	17.87	75
		SRR066648	3.90	15.22	75
Analysis of the transcriptome from S-deprived Chlamydomonas cells	WT strain D66 Sulfur repleted	SRR039669	1.4	6.91	35
		SRR039670	1.4	6.89	35
		SRR039671	1.2	6.33	35
		SRR039672	1.2	5.91	35
	WT strain D66 Sulfur deprived	SRR039673	1.3	6.74	35
		SRR039674	1.2	6.10	35
		SRR039675	1.4	7.22	35
	Ars11 mutant Sulfur repleted	SRR039676	0.77	4.22	33
		SRR039677	0.77	4.26	33
	Ars11 mutant Sulfur deprived	SRR039678	0.84	4.52	33
		SRR039679	0.89	4.80	33
