# Supplementary figures and images for: ChlamyNET: a Chlamydomonas gene co-expression network reveals global properties of the transcriptome and the early setup of key co-expression patterns in the green lineage
Source: BMC Genomics. 2016 Mar 12;17:227. doi: 10.1186/s12864-016-2564-y (PMC4788957; doi:10.1186/s12864-016-2564-y)

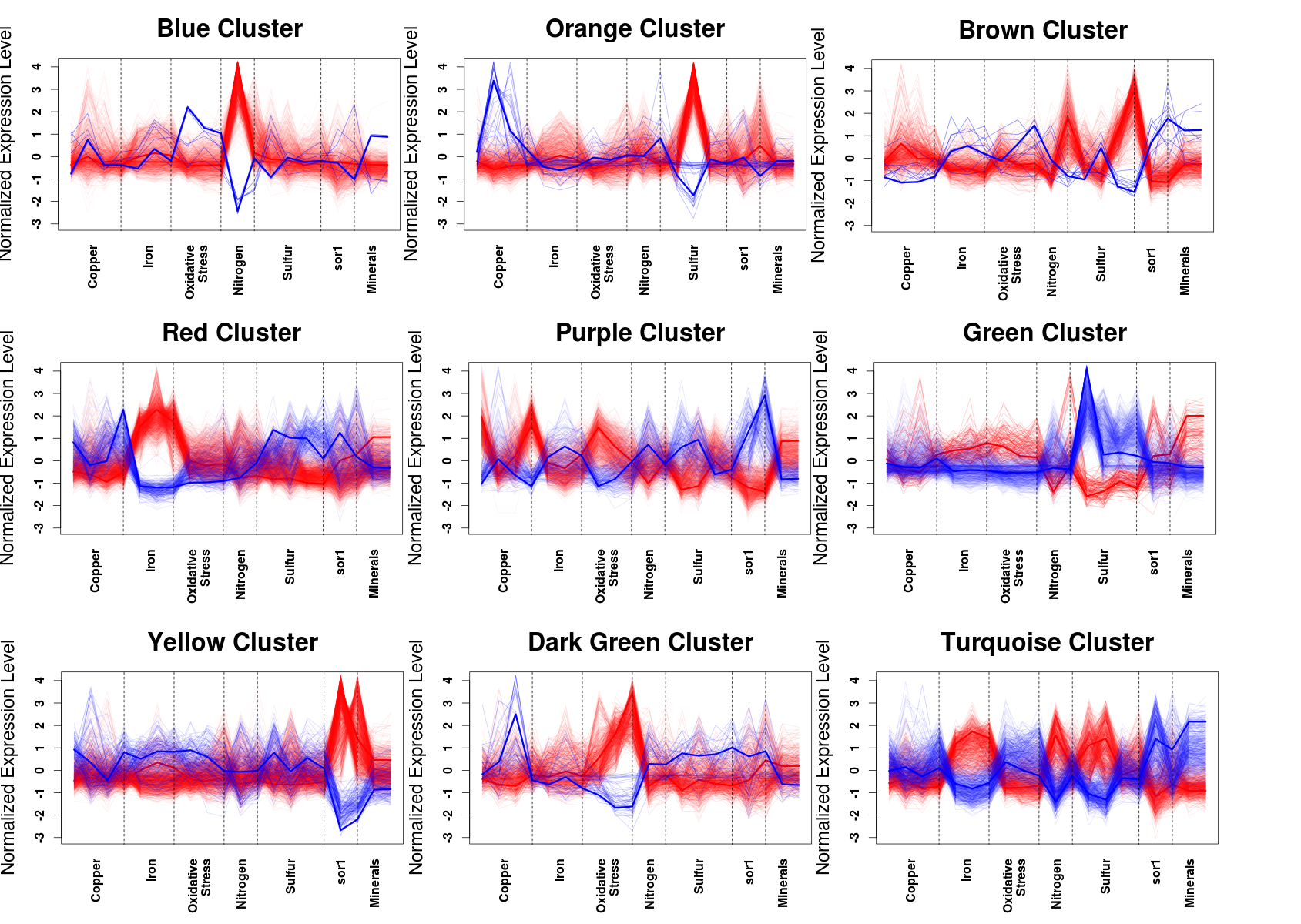

Supplement: Additional file 2: Figure S1. — Gene Cluster Expression Profiles. The normalized expression of every gene in each cluster is represented in grey. For each cluster the red line represents the mean positive expression profile whereas the blue line represents the mean negative expression profile. Recall that the absolute value of the correlation is used to define co-expression. Note that the expression profiles for each cluster are distinct. (TIFF 7917 kb) [file 12864_2016_2564_MOESM2_ESM.tiff]

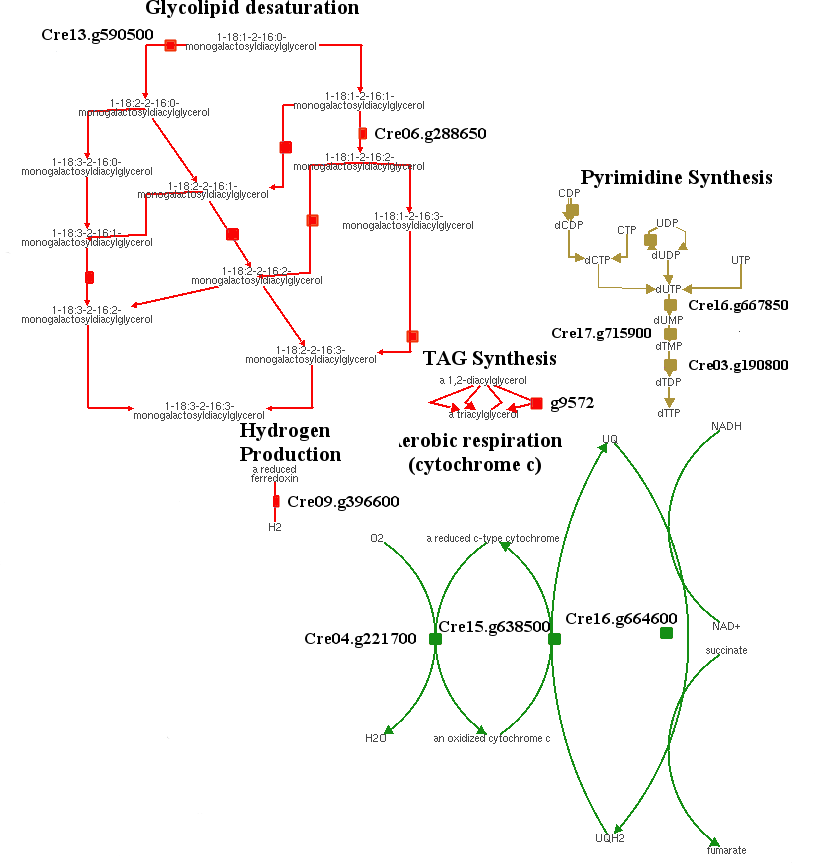

Supplement: Additional file 3: Figure S2. — Metabolic Pathways Contained in Clusters 2 (Brown), 3 (Red) and 7 (Green) from Figure 4. Analysis of the Chlamydomonas metabolic pathways revealed that cluster 2 (brown) in Figure 4 is enriched in metabolic processes involved in the DNA and RNA synthesis such as the pyrimidine synthesis pathways. Nevertheless, clusters 3 (red) and 7 (green) (Figure 4) were not so significantly enriched in metabolic pathways. Nevertheless, in cluster 3 (red), with a low significance, the synthesis pathway for triacylglycerol using galactolypids produced by glycolipid desaturation as acyl donors, was identified. In cluster 7 (green), the aerobic respiration pathway was fully included. (PNG 50 kb) [file 12864_2016_2564_MOESM3_ESM.png]

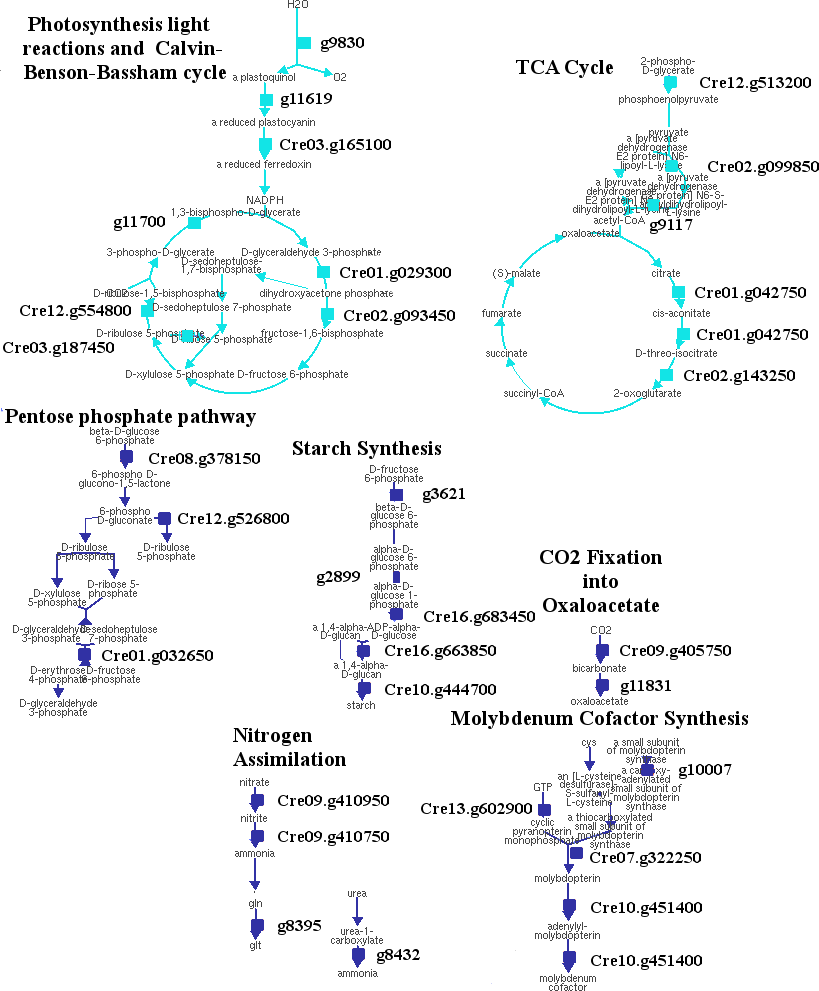

Supplement: Additional file 4: Figure S3. — Metabolic Pathways Contained in Clusters 9 (Blue) and 8 (Turquoise) from Figure 4. Analysis of the Chlamydomonas metabolic pathways revealed that cluster 9 (blue) and 8 (turquoise) (Figure 4) are enriched in diverse metabolic processes. Cluster 9 (blue) contains genes involved in core carbon/nitrogen metabolic pathways such as starch biosynthesis, the oxydative branch of the pentose phosphate pathway and nitrogen assimilation pathways co-expressed with the needed molybdenum cofactor synthetic pathway. Cluster 8 (turquoise), the two most important metabolic pathways in photosynthetic organisms, the Calvin cycle and the TCA cycle, have a significant number of genes represented. (PNG 98 kb) [file 12864_2016_2564_MOESM4_ESM.png]

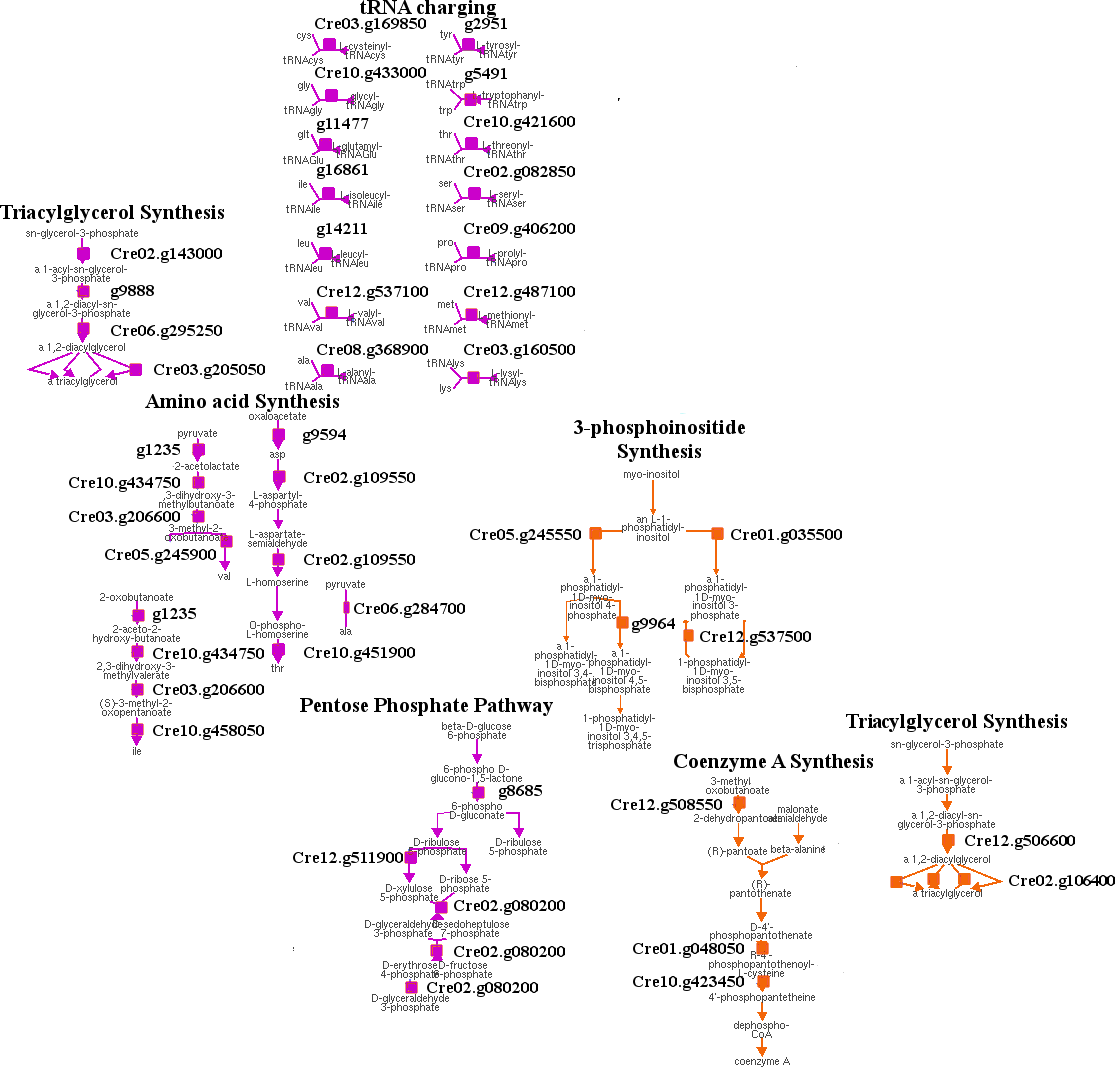

Supplement: Additional file 5: Figure S4. — Metabolic Pathways Contained in Cluster 1 (Orange) and 4 (Purple) from Figure 4. Analysis of the Chlamydomonas metabolic pathways revealed that cluster 1 (orange) and 4 (purple) (Figure 4) are enriched in specific metabolic processes. Cluster 4 (purple) comprises metabolic pathways associated with protein synthesis such as the tRNA charging pathways, aminoacid biosynthesis and the non-oxydative branch of the pentose phosphate pathway. The TAG biosynthetic pathway that preferentially uses as acyl donors galactolipids is also a member of this cluster. Cluster 1 (orange) is enriched in genes involved in lipid metabolism, including the TAG biosynthetic pathway which uses phospholipids that are in turn produced in the phosphatidylinositol pathway as acyl donors. The pathway for the synthesis of the necessary coenzyme A, is also present in this cluster. (PNG 119 kb) [file 12864_2016_2564_MOESM5_ESM.png]
