# Supplementary material for: ChlamyNET: a Chlamydomonas gene co-expression network reveals global properties of the transcriptome and the early setup of key co-expression patterns in the green lineage
Source: BMC Genomics. 2016 Mar 12;17:227. doi: 10.1186/s12864-016-2564-y (PMC4788957; doi:10.1186/s12864-016-2564-y)
Supplement: Additional file 6: Table S2. — Biological Processes Potentially Controlled by the Light-regulated TFs and TRs in ChlamyNET (PDF 8 kb) [file 12864_2016_2564_MOESM6_ESM.pdf]

GO term	Description	p value	Representative Genes
GO:0055085	transmembrane transport	9.8e-07	Cre08.g367150 Cre10.g453400 Cre12.g502600 Cre10.g445850 g16355 Cre09.g396000 Cre01.g009400 Cre02.g108550 Cre13.g564650 Cre10.g445050 Cre10.g448450 Cre16.g648300
GO:0006777	Mo-molybdopterin cofactor biosynthetic process	1.0e-06	Cre13.g602900 Cre07.g322250 g9002 Cre06.g282150 Cre10.g451400
GO:0005975	carbohydrate metabolic process	0.00299	Cre10.g458350 Cre01.g032650 Cre08.g384750 Cre07.g336950 Cre12.g552200 Cre01.g053000 g3160
GO:0006468	protein phosphorylation	0.01315	Cre02.g108650 Cre03.g168150 Cre08.g378950 g2226 g5041 Cre02.g087900 Cre07.g347000 Cre09.g413400
